# LRRK2 regulates ArfGAP1 membrane localization, activity and neuronal integrity via phosphorylation within its lipid-sensing ALPS2 motif

**DOI:** 10.3389/fnmol.2026.1786336

**Published:** 2026-04-21

**Authors:** Md. Shariful Islam, Valentin Cóppola-Segovia, Alessandra Musso, Darren J. Moore

**Affiliations:** 1 Department of Neurodegenerative Science, Van Andel Institute, Grand Rapids, MI, United States; 2 Laboratory of Molecular Neurodegenerative Research, Brain Mind Institute, Swiss Federal Institute of Technology (EPFL), Lausanne, Switzerland

**Keywords:** ArfGAP1, Golgi, LRRK2, mitochondria, phosphorylation

## Abstract

**Introduction:**

Mutations in the *leucine-rich repeat kinase 2* (*LRRK2*) gene cause late-onset, autosomal dominant Parkinson’s disease (PD). *LRRK2* encodes a multi-domain protein containing a Roc GTPase domain and a serine/threonine-directed protein kinase domain, with PD-linked mutations known to enhance LRRK2 kinase activity and neuronal toxicity. Our previous studies identified the Golgi protein, ADP-Ribosylation Factor GTPase-Activating Protein 1 (ArfGAP1), as a novel modifier of LRRK2-induced cellular toxicity, where it can serve as a GAP-like protein and a robust kinase substrate of LRRK2.

**Methods:**

Here, we further explore the phosphorylation of ArfGAP1 by LRRK2 and its functional consequences.

**Results:**

LRRK2 mediates the robust phosphorylation of ArfGAP1 *in vitro* within its lipid-sensing ALPS2 motif at residues Ser284, Thr291, and Thr292. We mutated these three candidate phosphorylation sites, either alone or combined, to create hydrophobic phospho-null or charged phospho-mimicking versions of ArfGAP1. We find that modulating ArfGAP1 phosphorylation at these sites impairs its normal capacity to induce Golgi fragmentation upon overexpression in neural cells. Blocking phosphorylation impairs ArfGAP1-induced neurite outgrowth inhibition in primary neurons and protects against the pathogenic effects of PD-linked G2019S LRRK2. ArfGAP1 interactome analysis in neural cells identifies 114 putative interacting proteins with a proportion of these localized to mitochondria, including the outer membrane proteins Voltage-Dependent Anion Channel (VDAC) 1–3. An ArfGAP1 triple phospho-mimic mutant displays an increased interaction with mitochondrial VDACs owing to the redistribution of ArfGAP1 from the *cis*-Golgi to the cytoplasm. Mimicking ArfGAP1 phosphorylation also blocks the formation of Golgi-derived vesicles following mild ER stress.

**Discussion:**

Our data provides evidence for a complex functional interaction between LRRK2 and ArfGAP1 that serves to regulate ArfGAP1 subcellular localization, protein interactions, activity and neuronal integrity via LRRK2-mediated phosphorylation of its membrane-binding ALPS2 motif. Our findings support additional validation of ArfGAP1 as a putative therapeutic target for modulating *LRRK2*-linked PD.

## Introduction

Parkinson’s disease (PD) is a common and progressive neurodegenerative movement disorder that is thought to result from a complex interplay between aging, genetic factors and environmental exposure (Kalia et al., 2015; Obeso et al., 2017). While PD typically occurs as a sporadic disease, 5–10% of cases occur in a familial manner with mutations in at least 20 genes identified to cause monogenic forms of PD (Blauwendraat et al., 2020). Among these familial cases, mutations in the *leucine-rich repeat kinase 2* (*LRRK2*) gene cause late-onset, autosomal dominant PD (Paisán-Ruíz et al., 2004; Zimprich et al., 2004). In addition, genome-wide association studies implicate common non-coding variation at the *LRRK2* locus in sporadic PD risk (Chang et al., 2017; Nalls et al., 2014), indicating that *LRRK2* represents a pleomorphic risk gene for PD. At least seven mutations in *LRRK2* are known to be pathogenic based upon clear segregation with disease in *LRRK2*-linked families, including N1437H, R1441C/G/H, Y1699C, G2019S, and I2020T, with G2019S representing the most frequent mutation (Aasly et al., 2010; Biskup and West, 2009; Cookson, 2015; Healy et al., 2008; Islam and Moore, 2017). LRRK2 therefore represents an important and promising therapeutic target for familial and sporadic PD.

LRRK2 belongs to the ROCO protein family and contains multiple domains including a central catalytic region consisting of Ras-of-Complex (Roc) GTPase and serine/threonine-directed protein kinase domains separated by a C-terminal-of-Roc (COR) linker region (Islam and Moore, 2017; Pfeffer and Alessi, 2025). LRRK2 can function as a kinase and GTPase, with the capacity for GTP-binding serving to regulate kinase activity (Islam and Moore, 2017). A number of substrates have been identified for LRRK2 kinase activity, with a subset of ∼14 Rab GTPases (i.e., Rab8A/10/12/29) being the best characterized to date especially within mammalian cells (Pfeffer and Alessi, 2025; Steger et al., 2016; Steger et al., 2017). Additional LRRK2 kinase substrates have also been identified mostly using *in vitro* assays, including ArfGAP1 (Stafa et al., 2012; Xiong et al., 2012), RPS15 (Martin et al., 2014), β-tubulin (Gillardon, 2009; Krumova et al., 2015), MARK1 (Krumova et al., 2015), NSF (Belluzzi et al., 2016), snapin (Yun et al., 2013), synaptojanin-1 (Islam et al., 2016), auxilin 1 (Nguyen and Krainc, 2018), RGS2 (Dusonchet et al., 2014), FoxO1 (Kanao et al., 2010), and Futsch (Islam et al., 2016; Lee et al., 2010). Familial PD mutations in LRRK2 tend to cluster within the Roc, COR and kinase domains. Notably, familial PD mutations share the capacity to enhance kinase activity, either directly via the kinase activation loop (G2019S, I2020T) or indirectly via the Roc-COR tandem domain (R1441C/G/H, Y1699C) presumably by impairing GTP hydrolysis and prolonging the GTP-bound state (Islam and Moore, 2017; Pfeffer and Alessi, 2025). Familial PD mutations also commonly inhibit neurite outgrowth and induce cell death in primary neuronal culture models via a kinase-dependent mechanism (Greggio et al., 2006; Islam and Moore, 2017; Smith et al., 2006; West et al., 2007). The GTPase and kinase activity of LRRK2 are both important for the development of PD and further understanding the accessory factors and substrates that regulate these activities will provide key insight for developing therapeutic strategies to attenuate LRRK2 activity in PD.

Our previous studies have identified ADP-ribosylation factor GTPase-activating protein 1 (ArfGAP1) as a novel modifier of LRRK2 activity and cellular toxicity. The deletion of *GCS1*, an ortholog of mammalian ArfGAP1, was originally identified as a suppressor of human LRRK2-induced cellular toxicity in yeast (Xiong et al., 2010). Subsequent studies revealed that ArfGAP1 gene silencing rescues the inhibition of neurite outgrowth induced by G2019S LRRK2 in rodent primary cortical neurons, whereas co-expression of wild-type LRRK2 and ArfGAP1 synergistically promotes neurite outgrowth deficits (Stafa et al., 2012; Xiong et al., 2012). ArfGAP1 expression alone can also reduce neurite length in a manner that depends on endogenous LRRK2 (Stafa et al., 2012). These data indicate the complex functional relationship between ArfGAP1 and LRRK2 and suggest that ArfGAP1 is critically required for mediating the pathogenic actions of mutant LRRK2. At the molecular level, ArfGAP1 physically interacts with LRRK2 and promotes its GTP hydrolysis activity and unexpectedly enhances LRRK2 kinase activity (Stafa et al., 2012; Xiong et al., 2012). ArfGAP1 also serves as a robust and direct substrate of LRRK2-mediated phosphorylation (Stafa et al., 2012; Xiong et al., 2012). Therefore, ArfGAP1 represents an intriguing accessory protein for LRRK2 that may function as a GAP-like protein to modulate LRRK2 activity and as a kinase substrate of LRRK2. We hypothesize that ArfGAP1 could mediate LRRK2-induced toxicity either by (i) serving upstream as a GAP-like protein to enhance the GTPase activity of LRRK2, or (ii) serving downstream as a substrate of LRRK2-dependent phosphorylation. We have elected to explore the role of ArfGAP1 phosphorylation as a potential mechanism, since available cryo-EM structural data suggests that dimeric LRRK2 is unlikely to accommodate or require GAPs and guanine nucleotide exchange factors (GEFs) for regulating Roc GTPase activity (Myasnikov et al., 2021; Zhu et al., 2023). As such, LRRK2 has instead been proposed to function as a non-canonical GTPase, specifically via a GTPase-activated-by-dimerization (GAD) mechanism (Deyaert et al., 2017; Wauters et al., 2019).

We and others have shown that LRRK2 can directly phosphorylate ArfGAP1 *in vitro* (Stafa et al., 2012; Xiong et al., 2012), but the impact of phosphorylation on ArfGAP1 cellular function remains to be determined. ArfGAP1 is known to function as a GAP for the Golgi-localized small GTPase Arf1, that mediates the dissociation of the COPI coatomer complex from Golgi-derived vesicles, a prerequisite for vesicle fusion with target compartments such as the endoplasmic reticulum (ER) (Cukierman et al., 1995; Donaldson and Jackson, 2011; Donaldson et al., 1992; Liu et al., 2005; Taylor et al., 1992). As such, overexpression of ArfGAP1 in mammalian cells induces the redistribution of the entire Golgi complex to the ER via vesicular intermediates (Klausner et al., 1992; Liu et al., 2005; Peters et al., 1995). As ArfGAP1 is required for LRRK2-induced neurite outgrowth inhibition, and ArfGAP1 silencing in neurons is generally well-tolerated (Stafa et al., 2012; Xiong et al., 2012), ArfGAP1 inhibition may offer an alternative therapeutic target for attenuating LRRK2 activity and toxicity in PD. Here, we identify candidate LRRK2-specific phosphorylation sites in ArfGAP1 and explore the effects of modulating these phosphorylation sites on its subcellular localization, Golgi morphology and sorting, neurite outgrowth and protein interactions. Our data further elucidates the functional interaction between LRRK2 and ArfGAP1, and the significance of ArfGAP1 phosphorylation.

## Results

### LRRK2 phosphorylates ArfGAP1 *in vitro* at Ser284 and Thr291 or Thr292

**FIGURE 1:**
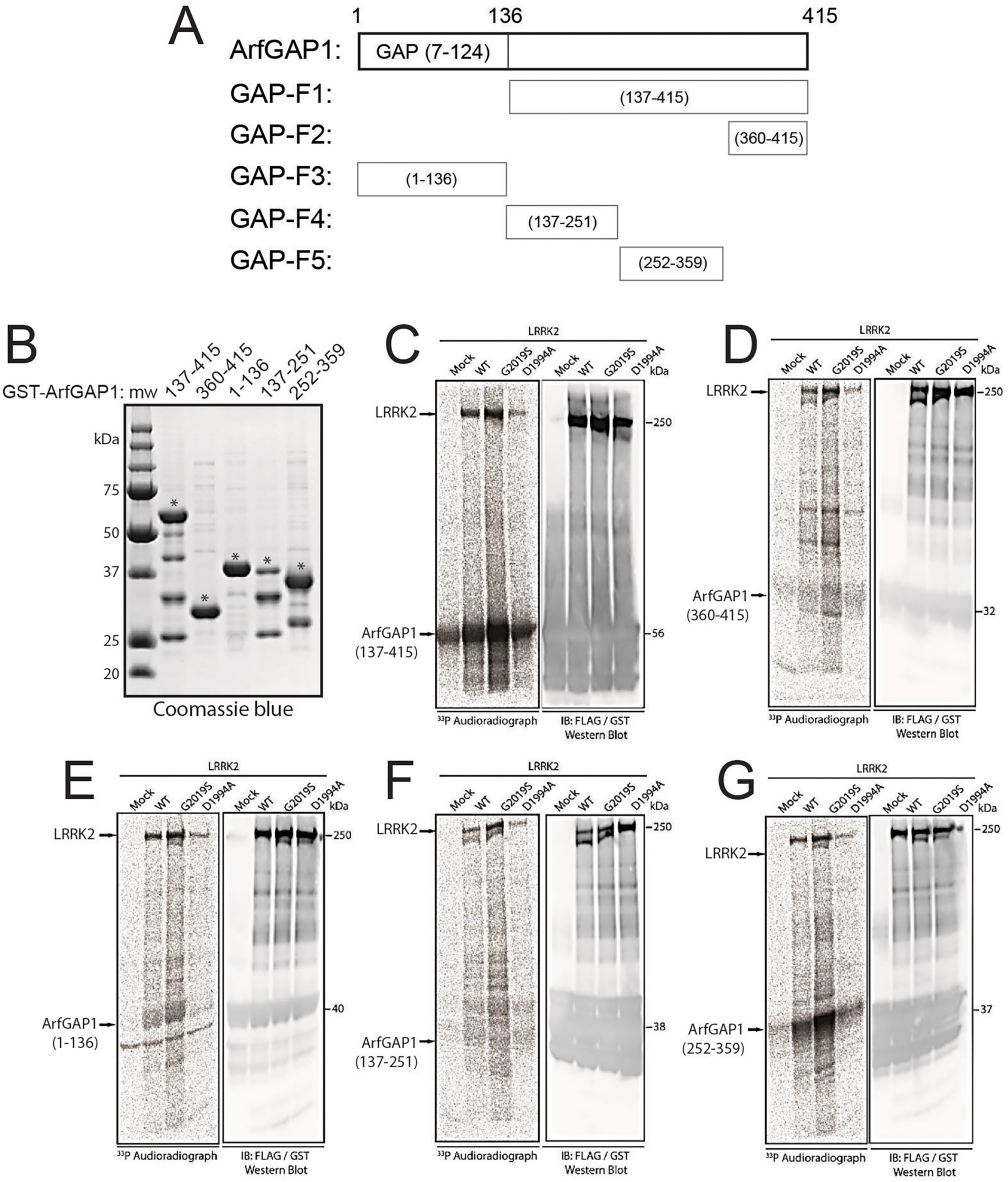
LRRK2-mediated phosphorylation of isolated ArfGAP1 domains. Schematic diagram of GST-tagged ArfGAP1 domain constructs (F1-F5, **A)** and their purity/levels on SDS-PAGE gels with Coomassie staining following purification using Glutathione-sepharose pull-down **(B)**. **(C–G)** Phosphorylation of each recombinant ArfGAP1 domain fragment by IP full-length human FLAG-tagged LRRK2 (WT, G2019S, D1994A) by *in vitro* kinase assay with [^33^P]-γ-ATP. Phosphorylation analysis of ArfGAP1 domains reveals that fragments 137–415 (F1, **C)** and 252–359 (F5, **G)** are abundantly phosphorylated by WT and G2019S LRRK2, with minimal phosphorylation of fragments 360–415 (F2, **D)**, 1–136 (F3, **E)** and 137–251 (F4, **F)**. LRRK2 autophosphorylation is also indicated with D1994A serving as a kinase-inactive control, and a mock FLAG IP (no LRRK2) serving as a control for non-specific incorporation of ^33^P. Indicated are representative ^33^P autoradiographs (left panel) and corresponding Western blots (right panel) co-probed with anti-GST (ArfGAP1) and anti-FLAG (LRRK2) antibodies to indicate equivalent protein loading. Molecular mass is indicated in kilodaltons (kDa). Positions of full-length LRRK2 and ArfGAP1 domains are indicated with arrows.

To explore the functional interaction between LRRK2 and ArfGAP1, we focused on defining the impact of LRRK2-mediated ArfGAP1 phosphorylation. We have previously demonstrated that ArfGAP1 serves as a robust substrate of LRRK2-mediated phosphorylation by *in vitro* radioactive kinase assay (Stafa et al., 2012). However, the identification and quantitation of LRRK2-specific ArfGAP1 phosphorylation sites was lacking in our previous work. A prior study identified six putative phosphorylation sites (S155, T189, T216, S246, S284, T292) within ArfGAP1 by mass spectrometry yet no quantitative analysis was provided, and it was unclear which LRRK2 variants were used in these assays, including whether comparison to a kinase-inactive LRRK2 negative control was included (Xiong et al., 2012). Furthermore, the simultaneous mutation of all six phospho-sites to alanine was required to inhibit LRRK2-mediated ArfGAP1 phosphorylation, suggesting that none of these six residues serve as a dominant phospho-site (Xiong et al., 2012). To initially locate the most abundant phosphorylation sites within ArfGAP1, we conducted *in vitro* radioactive kinase assays using [^33^P]-γ-ATP together with immunopurified recombinant full-length FLAG-tagged human LRRK2 (WT, G2019S or D1994A) and a series of GST-tagged ArfGAP1 protein fragments (residues 1–136, 137–415, 137–251, 252–359, and 360–415) (Figure 1A). We initially purified each GST-tagged ArfGAP1 protein from *E. coli* using glutathione-sepharose affinity columns and confirmed their relative purity and mass by SDS-PAGE and Coomassie staining (Figure 1B) before subjecting each protein fragment to *in vitro* kinase assays with LRRK2 (Figures 1C–G). In these kinase assays, we focused on the phosphorylation signal for purified GST-ArfGAP1 fragments of the expected size (denoted by *) rather than smaller truncated fragments that also copurified in some cases (i.e., fragments 137–415, 137–251, and 252–359; Figure 1B). We find that ArfGAP1 proteins containing residues 137–415 and 252–359 are robustly phosphorylated by G2019S LRRK2 and to a lesser extent by WT LRRK2, relative to kinase-inactive D1994A LRRK2 or in the absence of LRRK2 (Figures 1C,G). Other ArfGAP1 proteins (residues 1–136, 137–251, and 360–415) are only modestly phosphorylated by G2019S LRRK2 relative to other LRRK2 variants (Figures 1D–F). Importantly, the N-terminal catalytic GTPase-activating domain of ArfGAP1 (contained within residues 1–136), serves as a poor substrate of LRRK2 (Figure 1E), consistent with a prior study (Xiong et al., 2012). ArfGAP1 phosphorylation signal occurring in the absence of LRRK2 (mock) or in the presence of D1994A LRRK2 (Figures 1C–G), most likely results from non-specific phosphate incorporation or the potential presence of a contaminating kinase in the anti-FLAG IP samples used in these assays. These data suggest that the majority of LRRK2-mediated phosphorylation signal minimally resides within residues 252–359 of ArfGAP1 (GAP-F5 fragment), although additional less abundant sites are likely to exist outside of this region as suggested by higher phosphorylation of the GAP-F1 fragment (residues 137–415; Figure 1C).

**FIGURE 2:**
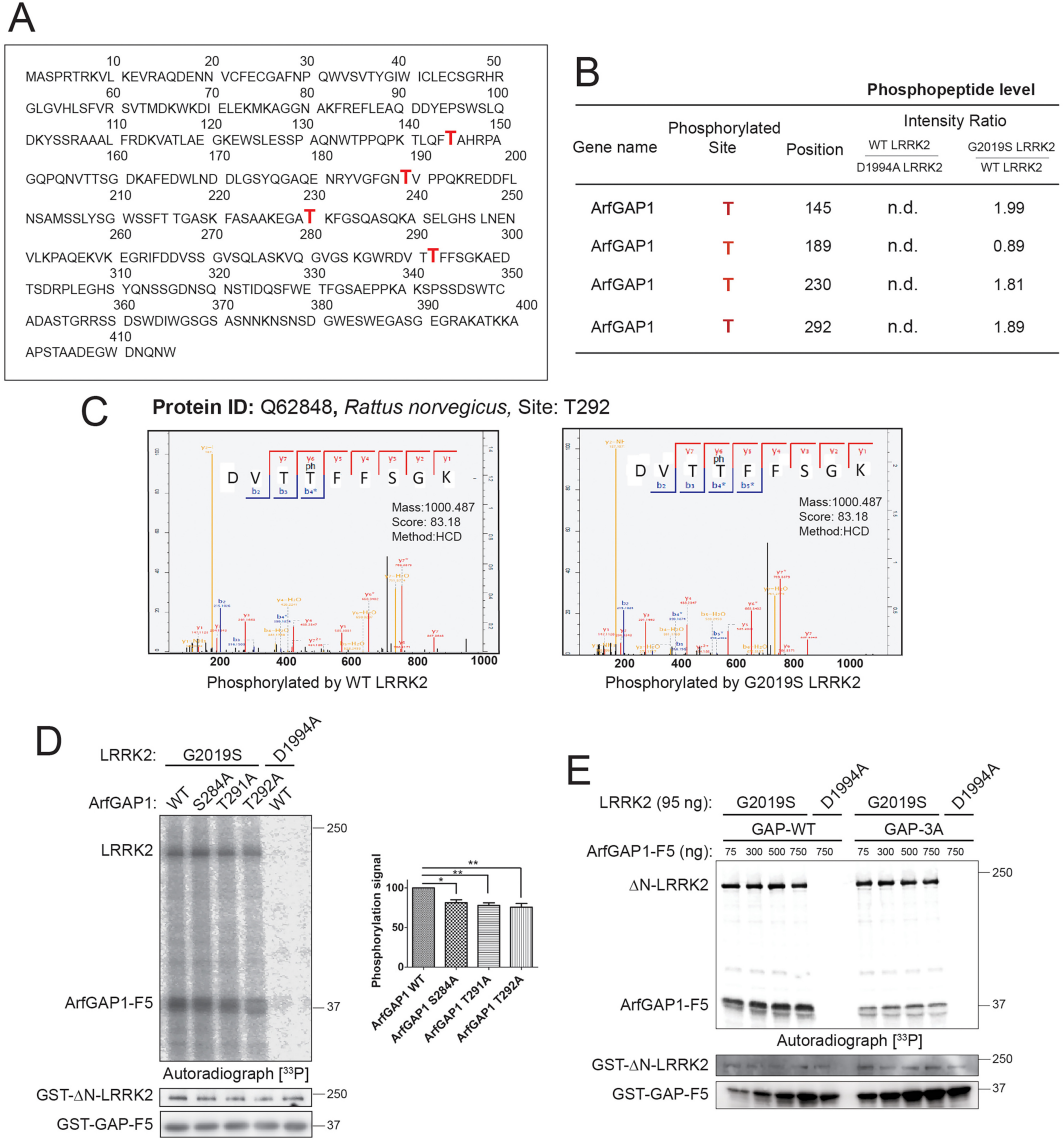
Identification of LRRK2-mediated ArfGAP1 phosphorylation sites. **(A)** Full-length protein sequence of rat ArfGAP1 with phosphorylated sites indicated in red. Mass spectrometry-based phospho-proteomic analysis identifies four major sites (T145, T189, T230, T292) that are phosphorylated by WT and G2019S LRRK2. **(B)** Table of significantly regulated ArfGAP1 phosphopeptides mediated by WT LRRK2 compared to kinase-inactive D1994A or G2019S LRRK2. pT145, pT230, and pT292 are increased ≥ 1.8-fold by G2019S relative to WT LRRK2. **(C)** Selected MS/MS spectra for ArfGAP1 phosphopeptide (DVTp**T**FFSGK; pT292) for WT or G2019S LRRK2. The respective phosphorylated site, the corresponding Uniprot ID and the Andromeda score are shown. **(D)** *In vitro* phosphorylation of GST-tagged ArfGAP1 domain (F5, residues 252–359) WT or single phospho-null mutants (S284A, T291A, T292A) by recombinant GST-tagged human LRRK2 (ΔN, residues 970–2,527, D1994A or G2019S) with [^33^P]-γ-ATP. Representative ^33^P autoradiograph indicating ArfGAP1 (and LRRK2) phosphorylation signal is shown, and Western blots probed with anti-GST antibody. Graph indicates densitometric analysis of each ArfGAP1-F5 phosphorylation signal (with G2019S LRRK2) normalized to total ArfGAP1-F5 levels, with bars representing the mean ± SEM (*n* = 3 experiments). *P < 0.05 or **P < 0.01 compared to WT ArfGAP1 signal by one-way ANOVA with Dunnett’s multiple comparisons test, as indicated. **(E)** *In vitro* phosphorylation of increasing quantities of GST-tagged ArfGAP1-F5 domain WT or triple phospho-null mutant (3A: S284A/T291A/T292A; 75–750 ng) by GST-tagged LRRK2 (D1994A or G2019S). Representative ^33^P autoradiograph and Western blots probed with anti-GST antibody are shown. Molecular mass is indicated in kilodaltons (kDa). Positions of ΔN-LRRK2 and ArfGAP1-F5 domain are indicated.

To identify the major phosphorylation sites in full-length ArfGAP1, we conducted similar *in vitro* kinase assays with non-radioactive ATP and subjected samples to mass spectrometry for quantitative analysis of ArfGAP1 phospho-peptides, comparing the effects of WT, G2019S and D1994A LRRK2. *In vitro* kinase assays with recombinant GST-tagged human LRRK2 variants (ΔN-LRRK2, residues 970–2,527) and full-length rat ArfGAP1 were subjected to in-solution digestion to generate peptide mixtures for nano-LC-MS/MS analysis (Q Exactive, Thermo Fisher) using a 2-h gradient time, as described (Williams et al., 2018). Mass spectrometry analysis of *in vitro* phosphorylated rat ArfGAP1 identifies four major sites (pT145, pT189, pT230, pT292) that are modified by LRRK2 to varying degrees (Figure 2A). We confirm the ∼2-fold enhanced phosphorylation of ArfGAP1 at pT145, pT230, and pT292 by G2019S LRRK2 relative to WT LRRK2, whereas pT189 levels are similar between WT and G2019S LRRK2 (Figure 2B). Importantly, kinase-inactive D1994A LRRK2 fails to detectably phosphorylate these four sites within ArfGAP1 (Figure 2B). Notably, we detect two threonine phosphorylation sites (pT189, pT292) identified in a prior study (Xiong et al., 2012), and two novel threonine sites (pT145, pT230), but we fail to detect four previously identified LRRK2-specific phosphorylation sites (pS155, pT216, pS246, pS284) in these assays (Xiong et al., 2012). Of note, rat ArfGAP1 contains a N155 residue instead of S155 present in the human and mouse protein. MS/MS spectra are shown for the most abundant ArfGAP1 phospho-peptide (^289^DVT[**pT**]FFSGK^297^) that supports phosphorylation at T292 in the presence of WT and G2019S LRRK2 (Figure 2C). Interestingly, of the combined phospho-sites identified by us and others, S155, T189, T230, S246, and S284 are known phosphorylation sites in mammalian ArfGAP1 according to the *PhosphoSitePlus*^®^ database, whereas intriguingly T291 rather than T292 is also a known phosphorylation site. Importantly, of the phospho-sites identified by MS, pT292 lies within the GAP-F5 fragment (residues 252–359) of ArfGAP1 that is robustly phosphorylated by LRRK2 (Figure 1G). We selected two additional putative ArfGAP1 phosphorylation sites (S284, T291) for further analysis, since pS284 was identified by a prior study (Xiong et al., 2012) and also lies within the GAP-F5 fragment, whereas the known phospho-site T291 is adjacent to T292 that makes it challenging to assign precise phospho-site localization within a single phospho-peptide.

To distinguish which of these three sites are the dominant phosphorylation site of ArfGAP1, we generated recombinant GST-tagged ArfGAP1 proteins (GAP-F5 fragment) harboring individual phospho-null alanine mutations (S284A, T291A, and T292A) for *in vitro* radioactive kinase assays with recombinant GST-tagged ΔN-LRRK2 (970–2,527). We find that each phospho-null mutation within the GAP-F5 protein significantly yet partially reduces G2019S LRRK2-mediated phosphorylation relative to WT GAP-F5, however, none of these three residues serve as a dominant phospho-site as they have equivalent effects (Figure 2D). D1994A LRRK2 fails to appreciably phosphorylate the WT GAP-F5 protein in this assay (Figure 2D). These data suggest that multiple sites within ArfGAP1, and particularly residues 252–359, are simultaneously phosphorylated by LRRK2. To further confirm these three ArfGAP1 phospho-sites, we generated a recombinant GST-tagged GAP-F5 protein containing all three phospho-null alanine mutations (referred to as 3A: S284A/T291A/T292A). The 3A mutation in GAP-F5 protein effectively blocks the increase in G2019S LRRK2-mediated phosphorylation in response to increasing amounts of GAP-F5 protein substrate, compared to the concentration-dependent increase in WT GAP-F5 phosphorylation, by *in vitro* radioactive kinase assay (Figure 2E). D1994A LRRK2 fails to phosphorylate the WT or 3A GAP-F5 proteins in these assays (Figure 2E), confirming the specificity of LRRK2 kinase activity for ArfGAP1. It is not possible to reliably determine whether T291 or T292 are phosphorylated by LRRK2 as alanine mutations of one site most likely impact the phosphorylation motif of the other site, whereas definitively resolving phosphorylation sites between adjacent identical residues (i.e., pTT or TpT) in a single phospho-peptide by mass spectrometry is not possible. In subsequent experiments, we have elected to mutate both T291 and T292 together to overcome this potential issue. Our attempts to verify these phospho-sites in cells co-expressing ArfGAP1 with LRRK2 variants combined with mass spectrometry analysis of immunoprecipitated ArfGAP1 were unsuccessful, most likely due to the low abundance and/or compartment-specific localization of these LRRK2-specific phosphorylation sites for detection in whole cell extracts. The development of ArfGAP1 phospho-specific antibodies is needed to confirm and localize these sites in different mammalian cell types. Collectively, our data suggests that S284 and T291 or T292 represent major candidate sites within ArfGAP1 that are phosphorylated by LRRK2 *in vitro*.

Interestingly, the S284 and T291/T292 phospho-sites are localized within a unique motif termed ArfGAP1 lipid-packing sensor 2 (ALPS2), that consists of residues 264–295 (Bigay et al., 2005; Mesmin et al., 2007). ArfGAP1 also contains a similar ALPS1 motif (residues 199–234), with both ALPS motifs localized to the non-catalytic C-terminal portion of ArfGAP1 relative to the GAP catalytic domain localized within the first 136 residues (Bigay et al., 2005; Mesmin et al., 2007). The ALPS 1 and 2 motifs bind preferentially to highly curved membranes and couple Arf1 GTP hydrolysis to COPI coat-induced membrane curvature (Bigay et al., 2005; Mesmin et al., 2007). The ALPS2 motif is normally unstructured but forms an amphipathic α-helix at the surface of highly curved membranes, with the α-helix containing an abundance of serine and threonine residues in its polar face (Mesmin et al., 2007). These Ser/Thr residues are critical for the sensitivity of the ALPS2 motif to membrane curvature (Mesmin et al., 2007). Notably, S284, T291, and T292 are incorporated within this α-helix of ALPS2, with the equivalent positions of S284 and T291 also being highly conserved in the ALPS1 motif (Mesmin et al., 2007). The pT230 site identified by mass spectrometry above also represents a highly conserved residue within ALPS1 (Mesmin et al., 2007). It is possible that LRRK2-dependent ArfGAP1 phosphorylation serves to regulate the sensitivity of the ALPS2 motif for binding with highly curved membranes, such as Golgi-derived vesicles, and therefore may modulate Arf1-dependent Golgi-to-ER retrograde transport.

### Impact of LRRK2-specific ArfGAP1 phosphorylation sites on Golgi complex integrity

**FIGURE 3:**
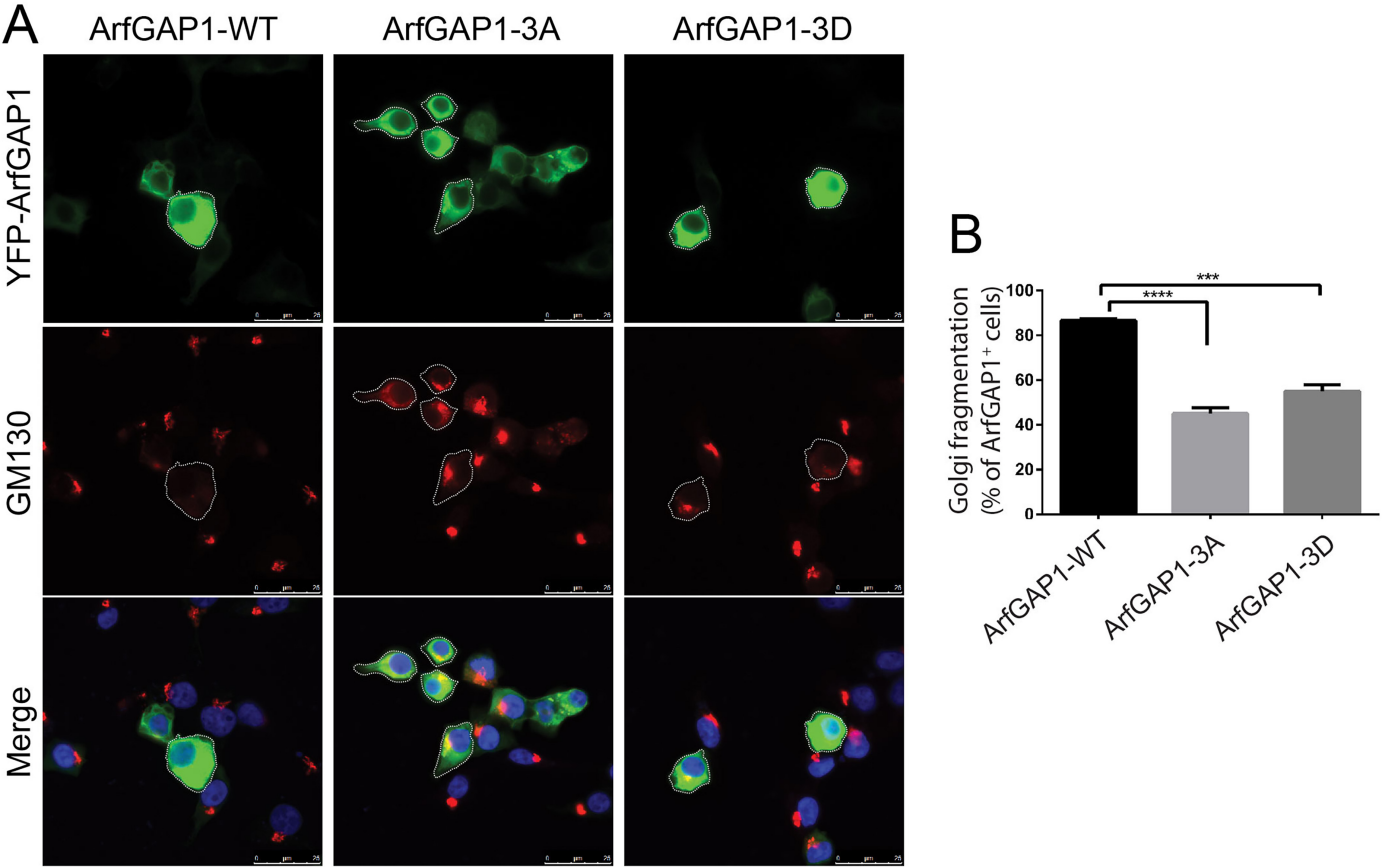
Phosphorylation mutants impair ArfGAP1-induced Golgi fragmentation in neural cells. **(A)** SH-SY5Y cells transiently expressing full-length YFP-tagged ArfGAP1 WT, triple phospho-null mutant (3A: S284A/T291A/T292A) or triple phospho-mimic mutant (S284D/T291D/T292D). Fixed cells were subjected to immunofluorescence with an antibody to the *cis*-Golgi marker, GM130. Fluorescence microscopy reveals a robust effect of ArfGAP1 overexpression on Golgi morphology, resulting in a normal (large perinuclear puncta), partially/fully fragmented (dispersed) or absent Golgi complex (white outline). Nuclear DAPI staining is also shown in merged images. **(B)** Quantitative analysis of Golgi fragmentation induced by ArfGAP1 overexpression (WT, 3A or 3D). Golgi fragmentation (dispersed or absent) is expressed as a percent of total YFP-ArfGAP1-positive cells for each variant. Bars represent mean ± SEM fragmented Golgi from 120 to 150 YFP-ArfGAP1-positive cells across at least three independent experiments/cultures. ***P < 0.001 and ****P < 0.0001 compared to WT ArfGAP1 by one-way ANOVA with Dunnett’s multiple comparisons test, as indicated.

ArfGAP1 is known to promote the GTP hydrolysis activity of Arf1 that is required for dissociation of the COPI coatmer complex from Golgi-derived vesicles, a prerequisite for vesicle fusion with target compartments such as the ER (Cukierman et al., 1995; Donaldson and Jackson, 2011; Liu et al., 2005). The overexpression of wild-type ArfGAP1 in mammalian cells leads to excessive Arf1 GTP hydrolysis that can impair Golgi complex integrity due to the increased redistribution of Golgi-derived vesicles to the ER (Klausner et al., 1992; Peters et al., 1995). To explore the impact of ArfGAP1 phosphorylation on this Arf1-dependent activity, we evaluated Golgi morphology in human SH-SY5Y neural cells overexpressing ArfGAP1 phospho-site variants by substituting either uncharged hydrophobic phospho-null (Alanine: A) or negatively-charged phospho-mimicking (Aspartic Acid: D) residues at Ser284, T291 and T292. Golgi morphology therefore serves as a useful functional readout of ArfGAP1 activity. Fluorescence microscopy reveals the effects of ArfGAP1 variants on Golgi morphology using the *cis*-Golgi marker GM130, resulting in either a normal intact perinuclear Golgi complex or more frequently an abnormally fragmented (or absent) Golgi complex (Figure 3A). Quantitative analysis of Golgi fragmentation indicates that overexpression of WT ArfGAP1 induces pronounced Golgi fragmentation in ∼85% of cells, whereas phospho-null (3A) ArfGAP1 significantly attenuates Golgi fragmentation (∼43% cells) (Figure 3B). Unexpectedly, phospho-mimic (3D) ArfGAP1 also similarly reduces Golgi fragmentation (∼55% cells) relative to WT ArfGAP1 (Figure 3B). Similar levels of Golgi complex fragmentation induced by WT ArfGAP1 expression are produced using a range of *cis*- or *trans*-Golgi markers, such as TGN46, Giantin or GOLGA4, similar to GM130 (Supplementary Figure S1). In addition, the analysis of individual ArfGAP1 phospho-null (S284A, T291A, or T292A) or phospho-mimic (S284D, T291D, or T292D) mutants alone does not reveal significant differences in Golgi fragmentation relative to WT ArfGAP1 (Supplementary Figure S1). We further demonstrate that a T291A/T292A double variant of ArfGAP1 induces an intermediate level of Golgi fragmentation relative to WT and 3A ArfGAP1 (Supplementary Figure S1). Collectively, our data suggests that the combined phosphorylation at S284 and T291 or T292 impairs the activity of ArfGAP1 to induce Arf1-mediated Golgi dispersal, whereas single (S284, T291, T292) mutations have no appreciable effect.

### Impact of LRRK2-specific ArfGAP1 phosphorylation sites on neurite outgrowth

**FIGURE 4:**
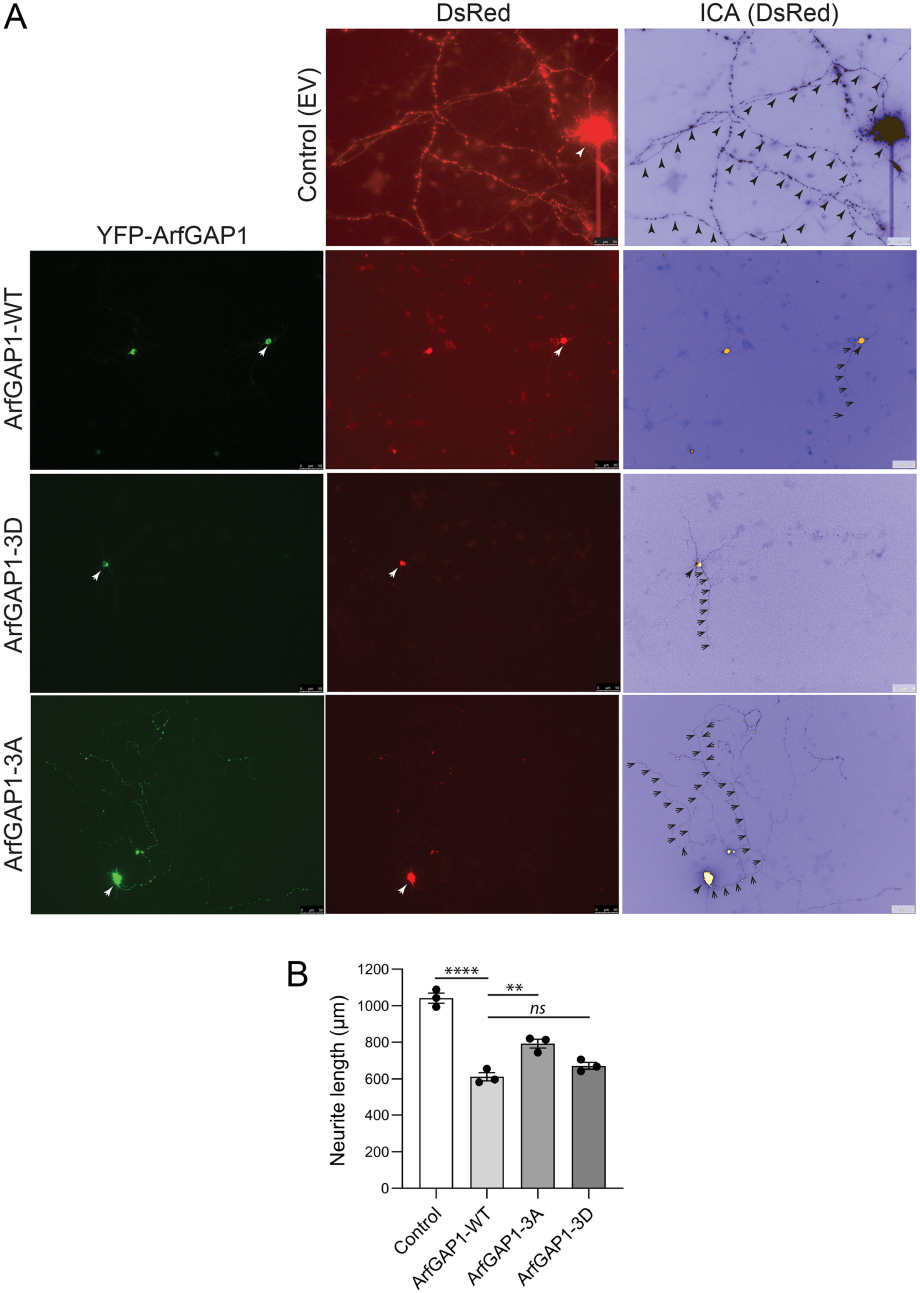
Preventing phosphorylation attenuates the ArfGAP1-induced inhibition of neurite outgrowth in cortical neurons. **(A)** Rat primary cortical neurons were co-transfected at DIV3 with YFP-ArfGAP1 (WT, 3A or 3D) or empty vector (control) and DsRed-Max-N1 plasmids, and fixed at DIV6 for confocal fluorescence microscopy analysis. Fluorescent images reveal the co-labeling of cortical neurons with YFP-ArfGAP1 (green) and DsRed (red), with the DsRed images pseudo colored (ICA) to enhance the contrast of neuritic processes. Neuronal soma (white arrows) and axonal processes (black arrowheads) are indicated. Scale bars: 50 μm. **(B)** Quantitative analysis of DsRed-positive axon length in YFP-ArfGAP1-positive neurons or control neurons (empty vector) is shown. Bars represent the mean ± SEM axon length (in μm) from 90 to 120 double DsRed-/YFP-ArfGAP1-positive neurons, or single DsRed-positive neurons (control), across three independent experiments/cultures (*n* = 3). **P < 0.005 or ****P < 0.0001 compared to ArfGAP1-WT by one-way ANOVA with Dunnett’s multiple comparisons test. *ns*, non-significant.

Mutant LRRK2 expression can inhibit neurite outgrowth in cultured primary cortical neurons, particularly axonal processes (Biosa et al., 2013; Stafa et al., 2012). We have previously shown that wild-type ArfGAP1 overexpression can similarly inhibit axonal outgrowth and reduce axonal length, whereas ArfGAP1 gene silencing can reverse the effects of G2019S LRRK2 on neurite length (Stafa et al., 2012). These data indicate that ArfGAP1 plays a role in regulating neurite outgrowth and potentially in neuronal integrity and viability. To evaluate the effects of LRRK2-specific ArfGAP1 phosphorylation sites on neurite outgrowth, we conducted quantitative assays by assessing axonal length in cultured rat primary cortical neurons. Cultures at days-*in-vitro* (DIV) 3 were transiently co-transfected with YFP-tagged ArfGAP1 variants or empty vector and pDsRed-Max-N1 (to mark neuronal processes) at a 10:1 molar ratio and fixed at DIV 6. Fluorescence microscopy was used to measure the longest DsRed-positive neurite (i.e., the axon) from YFP+ (ArfGAP1) or YFP- (empty vector) cortical neurons, as previously described (Stafa et al., 2012). We find that neurons overexpressing WT ArfGAP1 reveal a marked reduction in neurite length relative to control (empty vector) neurons, whereas unexpectedly, single ArfGAP1 phospho-null mutants (S284A, T291A or T292A) also markedly reduce neurite length to levels similar to WT ArfGAP1 (Supplementary Figure S2). Furthermore, we find that expression of single ArfGAP1 phospho-mimic mutants (S284D, T291D or T292D) also markedly reduces neurite length similar to WT ArfGAP1 (Supplementary Figure S3). These neurite length data are consistent with the lack of effect of single phospho-site mutants on ArfGAP1-induced Golgi fragmentation (Supplementary Figure S1). Accordingly, we compared the effects of triple ArfGAP1 phospho-site mutants (3A or 3D) on neurite length. Remarkably, we find that the 3A mutant partially yet significantly attenuates the impact of ArfGAP1 expression on neurite length relative to the WT or 3D proteins which are similar (Figure 4). Together, these data indicate that blocking the combined phosphorylation of ArfGAP1 at Ser284, T291 and T292 (3A mutant), but not at single sites, impairs neurite outgrowth inhibition consistent with reducing ArfGAP1 activity. Mimicking ArfGAP1 phosphorylation at these sites is sufficient for maximal inhibition of neurite outgrowth, similar to WT ArfGAP1, potentially suggesting that these phosphorylation sites are already saturated in the WT protein in cortical neurons.

### Blocking ArfGAP1 phosphorylation rescues G2019S LRRK2-induced neurite outgrowth inhibition

**FIGURE 5:**
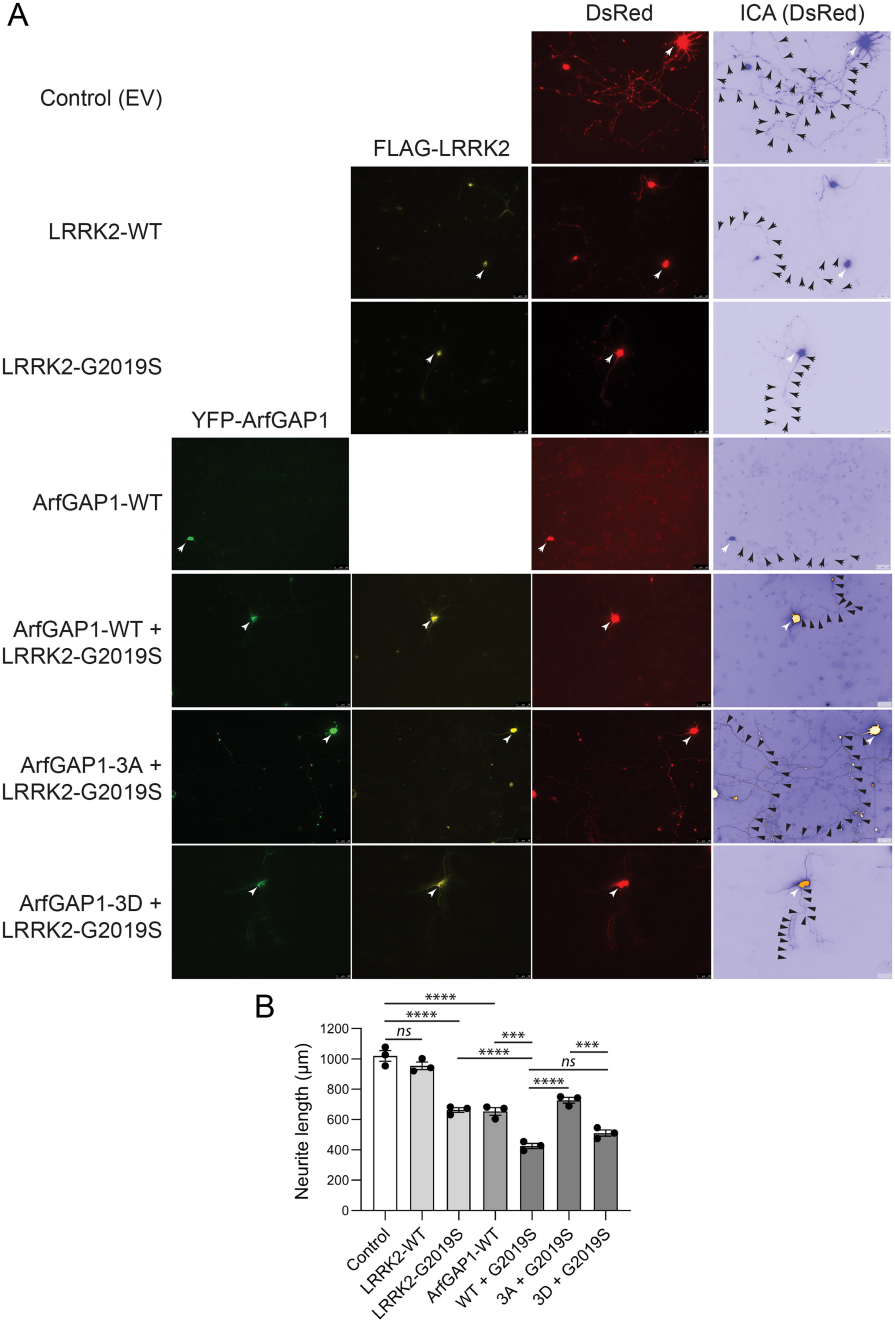
Preventing ArfGAP1 phosphorylation protects against the inhibition of neurite outgrowth induced by G2019S LRRK2. **(A)** Rat primary cortical neurons were co-transfected at DIV3 with combinations of YFP-ArfGAP1 (WT, 3A or 3D), FLAG-LRRK2 (WT or G2019S) and DsRed-Max-N1 plasmids, and fixed at DIV6 for immunofluorescence analysis with anti-FLAG antibody. Fluorescent images reveal the co-labeling of cortical neurons with YFP-ArfGAP1 (green), G2019S LRRK2 (yellow) and DsRed (red), with the DsRed images pseudo colored (ICA) to enhance the contrast of neuritic processes. Neuronal soma (white arrows) and axonal processes (black arrowheads) are indicated. Scale bars: 50 μm. **(B)** Quantitative analysis of DsRed-positive axon length in YFP-ArfGAP1-/LRRK2-positive neurons, or ArfGAP1- and LRRK2-positive neurons alone, is shown. Bars represent the mean ± SEM axon length (in μm) from 90 to 120 triple DsRed-/YFP-ArfGAP1-/LRRK2-positive or double DsRed-/LRRK2- and DsRed-/ArfGAP1-positive neurons across three independent experiments/cultures (*n* = 3). ***P < 0.001 or ****P < 0.0001 between groups as indicated by one-way ANOVA with Tukey’s multiple comparisons test. *ns*, non-significant.

We have shown that the co-expression of ArfGAP1 and LRRK2 has a synergistic effect on reducing neurite length that is dependent in part on normal LRRK2 GTPase activity (Stafa et al., 2012). To evaluate the impact of ArfGAP1 phosphorylation sites on the interaction with LRRK2, we co-transfected cortical neurons with a combination of FLAG-tagged LRRK2 (WT or G2019S), YFP-ArfGAP1 variants (WT, 3A, or 3D) and pDsRed-Max-N1 plasmids at a 10:10:1 molar ratio. Initially, we find that expression of G2019S LRRK2 or WT ArfGAP1 alone markedly and equivalently reduce neurite length by 35–40% compared to WT LRRK2 or control (empty vector) neurons (Figure 5). The co-expression of WT or 3D ArfGAP1 with G2019S LRRK2 reduces neurite length by 50–60% compared to control neurons, whereas surprisingly the phospho-null 3A mutant lacks a synergistic effect with G2019S LRRK2 revealing only a 20–25% reduction of neurite length compared to control neurons (Figure 5). Collectively, these data suggest that ArfGAP1 phosphorylation at these sites is required and sufficient for the synergistic interaction with G2019S LRRK2 in reducing neurite length, and blocking these sites (in the 3A mutant) can interfere with the neurite outgrowth deficits induced by G2019S LRRK2.

### ArfGAP1 interactome analysis identifies proteins localized to the Golgi, mitochondrial, and cytoplasmic compartments

**FIGURE 6:**
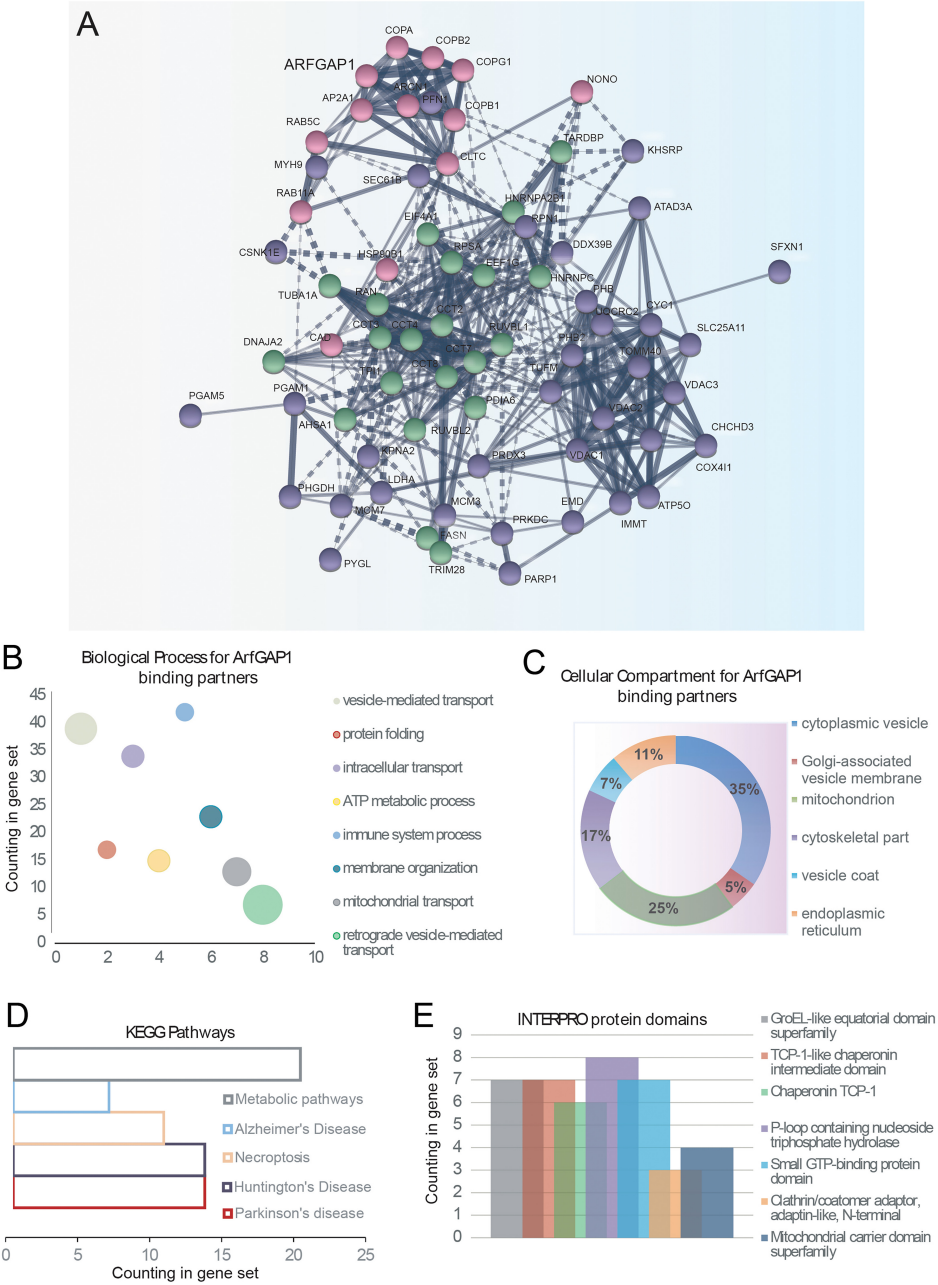
Protein interactome of ArfGAP1 reveals binding to a collection of mitochondrial proteins in neural cells. SH-SY5Y cells transiently expressing wild-type YFP-tagged ArfGAP1, or empty plasmid as a control, were subjected to immunoprecipitation with an anti-GFP antibody and subjected to LC-MS/MS analysis. **(A)** K-means clustering of ArfGAP1 interaction maps of binding proteins identified by MS. Candidate interacting proteins (114) are segregated into three distinct clusters based on subcellular location: mitochondrial membrane (purple), cytoplasmic part (green) and Golgi membrane vesicle (pink). **(B,C)** Gene Ontology (GO) analysis of ArfGAP1-interacting partners. **(B)** GO biological process indicates ∼40 interacting proteins involved in “vesicle-mediated transport,” whereas **(C)** Pie chart represents the GO cellular compartments of all binding partners. **(D)** KEGG pathways associated with ArfGAP1 interactors. **(E)** INTERPRO protein domain analysis of ArfGAP1 interactors.

Since LRRK2-specific ArfGAP1 phospho-sites are localized within the non-catalytic domain, and specifically the ALPS2 motif, and regulate ArfGAP1-induced Golgi dispersal and neurite outgrowth deficits, we next sought to explore the protein interactome of ArfGAP1 and the potential impact of phosphorylation. Large-scale protein-protein interaction studies serve an important role in understanding the biological processes and cellular functions of a protein that are largely dependent on its subcellular localization and activity. ArfGAP1 can cycle between the Golgi complex, Golgi-derived vesicles and the cytosol (Liu et al., 2005), with phosphorylation impacting its actions at the Golgi. Accordingly, we first explored the interaction partners of ArfGAP1 in human cells by mass spectrometry. Detergent-soluble extracts derived from SH-SY5Y neural cells transiently expressing YFP-tagged wild-type ArfGAP1 were subjected to co-immunoprecipitation (IP) assays using anti-GFP antibody to enrich for interacting partners of ArfGAP1 and submitted for LC-MS/MS analysis. Similar anti-GFP IPs from cell extracts transfected with an empty plasmid served as a control to identify non-specific binding proteins that were subsequently excluded. MS-based proteome analysis with label-free protein quantification of biological triplicate experiments identifies 114 putative interacting proteins (FDR < 0.05, fold change ≥ 4) including several known ArfGAP1-interacting partners such as adaptor protein complex AP-2 subunit alpha-1 (AP2A1) (Rawet et al., 2010) and COPI coatomer subunits (COPA, COPB1, COPB2, COPG1) (Eugster et al., 2000; Figure 6A and Supplementary Table S1). The interactome screen also identifies several novel ArfGAP1 interactors, such as the mitochondrial proteins VDAC1, VDAC2, VDAC3 and TOMM40, as well as Rab1B, Rab5C and Rab11A that are localized to intracellular vesicular membranes (Figure 6A and Supplementary Table S1). Surprisingly, we do not identify Arf1 or LRRK2 in our interactome dataset, potentially due to their labile interaction and/or low abundance in SH-SY5Y cells.

To explore the intermolecular connections between all identified binding partners, we integrated Search Tool for Retrieval of Interacting Genes (STRING) analysis with our MS-based ArfGAP1 interactome data. The KMEANS clustering in protein-protein interaction networks identifies three distinct clusters, (i) mitochondrial membrane, (ii) cytoplasmic part, and (iii) Golgi membrane vesicle, suggesting that ArfGAP1-interacting partners generally segregate into these three subcellular compartments (Figure 6A). To derive additional insight from our interactome data, Gene Ontology (GO) analysis was conducted to describe the biological processes and cellular compartments of ArfGAP1-interacting partners. For GO biological processes, 39 proteins are linked to vesicle-mediated transport, whereas there are also 6 proteins (COPA, COPB1, COPB2, COPG1, ARCN1, and Rab1B) specifically linked to retrograde vesicle-mediated transport (Figure 6B). For GO cellular compartments, most interactors (35%) are cytoplasmic vesicle proteins with only 5% of proteins localizing to Golgi-associated vesicle membranes, whereas 25% of proteins are associated with mitochondria (Figure 6C). Mitochondrial interactors include proteins localized to the outer membrane (i.e., VDAC1-3, TOMM40), inner membrane/cristae (i.e., SLC25A3/5/6/11, ATP5O, CYC1), intermembrane space (i.e., CHCHD3), and matrix (i.e., PRDX3) compartments (Supplementary Table S1). We also performed pathway analysis on the ArfGAP1 interactome using the KEGG Pathways database. Notably, this analysis identifies “PD pathways” along with four other pathways including “Huntington’s disease” and “Alzheimer’s disease” (*P* < 0.05) (Figure 6D), and there are several interacting partners, such as VDAC1-3 that are directly linked with PD pathways (Sun et al., 2012). We further attempted to classify interacting proteins into protein families to potentially identify key domains within the ArfGAP1-interacting protein network. INTERPRO analysis predicts several protein domains, including the mitochondrial carrier domain superfamily, P-loop containing nucleoside triphosphate hydrolase, and small GTP-binding protein domains, that are shared by at least 20 interacting partners (Figure 6E). Of note, five interactors contain a small GTP-binding protein domain (Rab1B, Rab5C, Rab11A, Ran, and EEF1G). Taken together, our comprehensive MS-based interactome data provides a global view of ArfGAP1 interaction networks and also provides a strong connection to mitochondrial membranes.

### ArfGAP1 phosphorylation impacts its subcellular localization and interaction with VDACs

**FIGURE 7:**
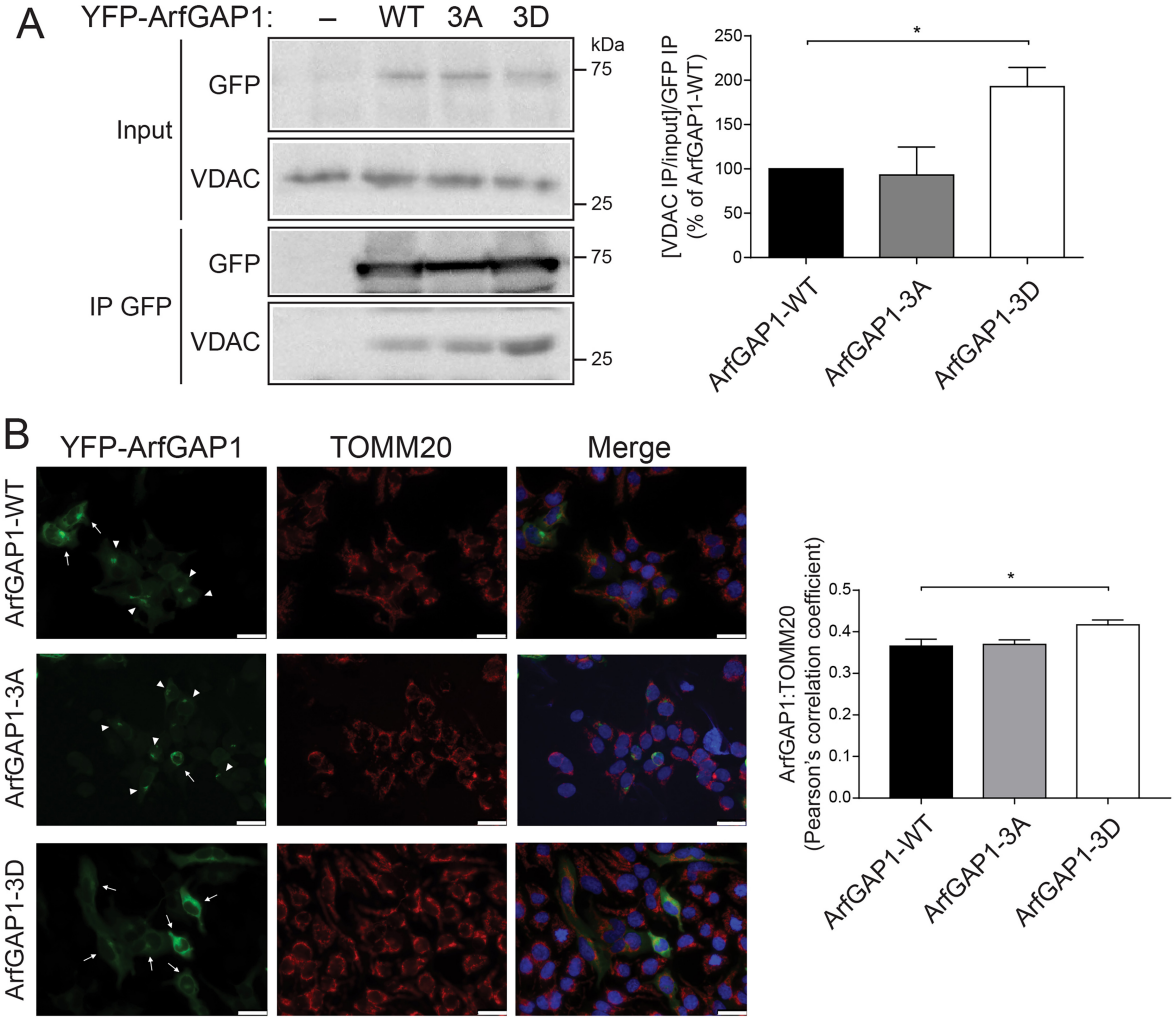
Phospho-mimic mutations increase ArfGAP1 binding to VDACs and its mitochondrial localization. **(A)** SH-SY5Y cell extracts transiently expressing YFP-ArfGAP1 (WT, 3A or 3D) or empty plasmid were subjected to immunoprecipitation (IP) with anti-GFP antibody, and IP and input lysates were probed by Western blotting with anti-pan-VDAC1-3 and anti-GFP antibodies. Graph indicates densitometric analysis of VDAC binding for each ArfGAP1 variant, with data expressed as the ratio of VDAC IP levels normalized to input VDAC levels, and these ratios further normalized to the level of each IP ArfGAP1 variant. Bars represent mean ± SEM (*n* ≥ 3 independent experiments). Molecular mass is indicated in kilodaltons (kDa). **(B)** SH-SY5Y cells expressing YFP-ArfGAP1 (WT, 3A or 3D) subjected to immunofluorescence with anti-TOMM20 antibody. Representative fluorescent microscopic images indicate partial colocalization of mitochondrial TOMM20 (red) and YFP-ArfGAP1 (green). Nuclear DAPI (blue) is shown in merged images. Arrows indicate diffuse localization of YFP-ArfGAP1 (mainly 3D mutant) and arrowheads indicate perinuclear Golgi localization (mainly WT and 3A mutant). Scale bars: 25 μm. Graph indicates Pearson’s correlation coefficients for ArfGAP1/TOMM20 colocalization. Bars represent mean ± SEM (*n* ≥ 3 independent experiments). *P < 0.05 compared to WT ArfGAP1 by one-way ANOVA with Dunnett’s multiple comparisons test, as indicated.

**FIGURE 8:**
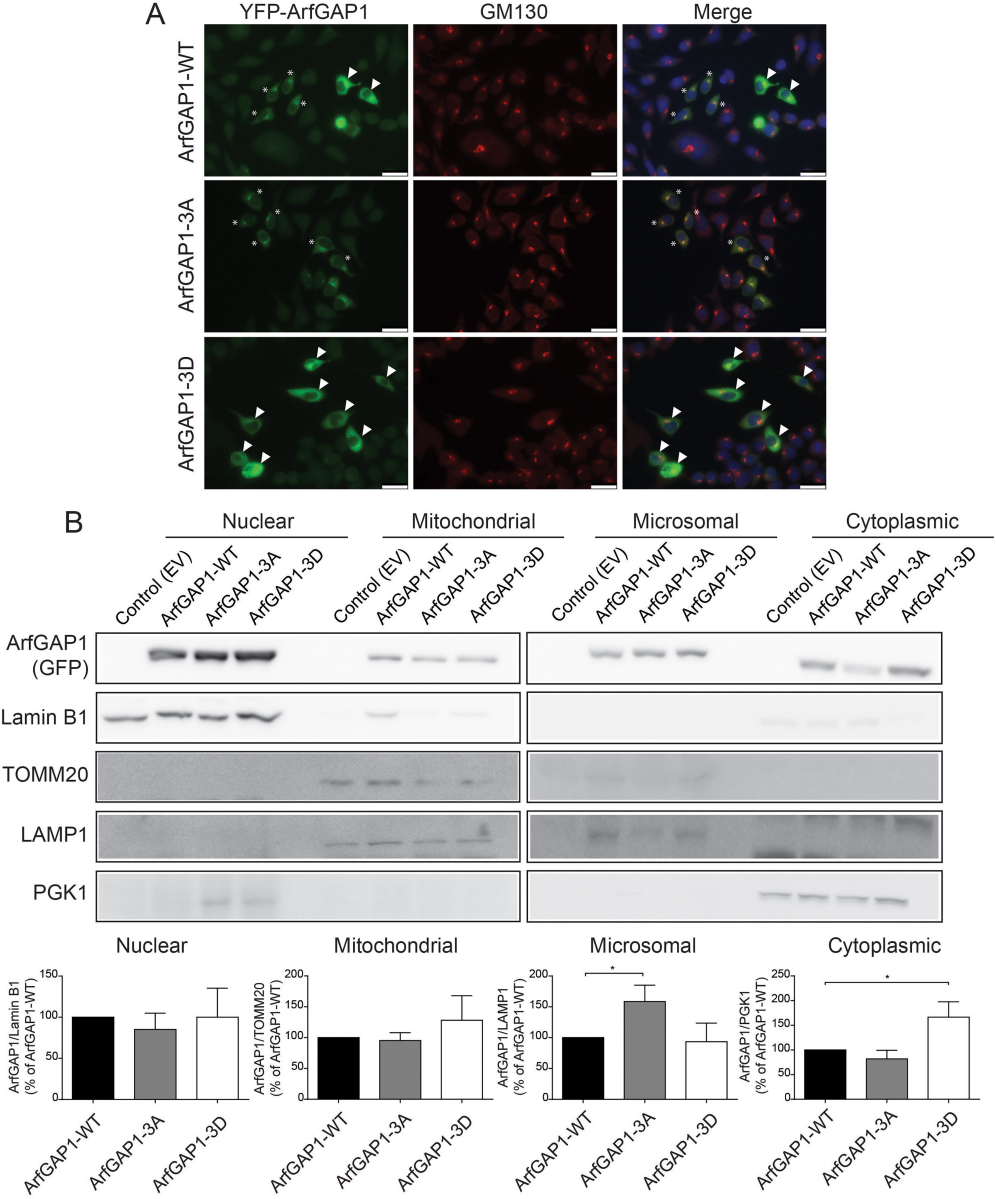
Phospho-mimic mutations disrupt the localization of ArfGAP1 to the *cis*-Golgi and drive a cytoplasmic location. **(A)** SH-SY5Y cells expressing YFP-ArfGAP1 (WT, 3A or 3D) subjected to immunofluorescence with anti-GM130 antibody. Representative fluorescent microscopic images indicate colocalization of *cis*-Golgi GM130 (red) and YFP-ArfGAP1 (green). Nuclear DAPI (blue) is shown in merged images. Asterisks indicate perinuclear Golgi localization of YFP-ArfGAP1 (3A mutant > WT) and arrowheads indicate diffuse cytoplasmic localization (3D mutant > > WT). Scale bar: 25 μm. **(B)** Subcellular fractionation by differential centrifugation of SH-SY5Y cell extracts expressing YFP-ArfGAP1 (WT, 3A or 3D) or control empty vector. Western blot analysis of nuclear, mitochondrial, microsomal and cytoplasmic fractions with anti-GFP antibody and antibodies to markers enriched in each fraction (Lamin B1/nuclear, TOMM20/mitochondria, LAMP1/microsomes, PGK1/cytoplasmic). ArfGAP1 variants are broadly detected across the different subcellular fractions, with subtle differences. Graphs indicate densitometric analysis of YFP-ArfGAP1 levels in each subcellular fractionation normalized to their respective compartment marker. Bars represented the mean ± SEM levels of ArfGAP1 (*n* ≥ 3 independent experiments). *P < 0.05 compared to WT ArfGAP1 by one-way ANOVA with Dunnett’s multiple comparisons test, as indicated.

From the ArfGAP1 interactome analysis, the robust connection to mitochondrial membranes was both intriguing and unappreciated in prior studies. As such, voltage-dependent anion channels (VDAC) 1–3, that are localized to the outer mitochondrial membrane, emerged as interesting and novel interacting partners of ArfGAP1. We elected to evaluate whether ArfGAP1 phosphorylation influences the interaction with VDACs by conducting co-IP assays with anti-GFP antibody from SH-SY5Y cell extracts transiently expressing YFP-tagged ArfGAP1 variants (WT, 3A or 3D). Intriguingly, the phospho-mimic 3D mutant markedly increases the interaction between ArfGAP1 and VDACs (using a pan-VDAC1-3 antibody) compared to WT or 3A ArfGAP1 (Figure 7A). As VDACs are integral transmembrane proteins that traverse the outer mitochondrial membrane (Ponnalagu and Singh, 2017), we hypothesized that the ArfGAP1-3D variant is in close proximity to mitochondria. We therefore conducted confocal fluorescence microscopy on SH-SY5Y cells transiently expressing YFP-ArfGAP1 variants together with the outer mitochondrial membrane marker, TOMM20. Correlation analysis reveals a significant increase in the colocalization of ArfGAP1-3D with mitochondrial TOMM20, compared to WT or 3A ArfGAP1 (Figure 7B). While ArfGAP1 is predominantly a Golgi-localized protein (Parnis et al., 2006; Pevzner et al., 2012; Yu and Roth, 2002), ArfGAP1-3D is mostly cytoplasmic whereas WT and 3A ArfGAP1 adopt a more typical perinuclear localization (Figure 7B; Parnis et al., 2006; Pevzner et al., 2012; Yu and Roth, 2002). As the C-terminal domain is involved in regulating the subcellular localization of ArfGAP1 and its interaction with membranes (Parnis et al., 2006; Yu and Roth, 2002), particularly via its ALPS 1 and 2 motifs, we asked whether the phospho-null and phospho-mimic variants adopt different localizations. Confocal fluorescence analysis of SH-SY5Y cells expressing YFP-ArfGAP1 variants reveals that WT ArfGAP1, and to a greater extent the 3A mutant, mostly co-localize with the GM130-labeled Golgi complex, whereas ArfGAP1-3D adopts a distinct cytoplasmic-like localization (Figure 8A). To confirm this differential localization of the phospho-mimic 3D mutant, we performed subcellular fractionation analysis on SH-SY5Y cells expressing YFP-ArfGAP1 variants. We detect WT ArfGAP1 broadly distributed across all subcellular fractions, with localization to nuclear, microsomal (i.e., Golgi), cytoplasmic and mitochondrial fractions (Figure 8B). We find that ArfGAP1-3A is significantly enriched in the microsomal fraction, whereas ArfGAP1-3D is significantly enriched in the cytoplasmic fraction, compared to WT ArfGAP1 (Figure 8B). ArfGAP1 variants display similar levels of enrichment in the nuclear and mitochondrial fractions (Figure 8B). These fractionation data confirm the differential localization of 3A and 3D ArfGAP1 mutants indicated by immunofluorescence analysis (Figure 8A) and suggest that the phospho-null 3A mutant drives a Golgi localization whereas the phospho-mimic 3D mutant drives a cytoplasmic localization. Collectively, our data suggests that phosphorylation at these *in vitro* candidate sites regulates ArfGAP1 subcellular localization between the Golgi and cytoplasmic compartments, and the interaction with outer mitochondrial membrane proteins such as VDACs.

### ArfGAP1 phosphorylation regulates the formation of Golgi-derived vesicles induced by endoplasmic reticulum (ER) stress

**FIGURE 9:**
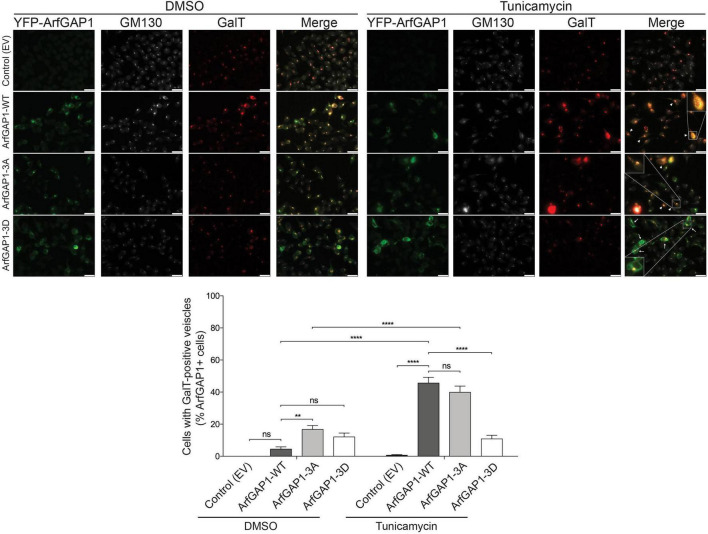
Phospho-mimic mutations disrupt ArfGAP1-induced Golgi-derived vesicle formation under mild ER stress. SH-SY5Y cells transiently co-expressing YFP-ArfGAP1 (WT, 3A, 3D) and mCherry-GalT were treated with vehicle (DMSO) or tunicamycin (2.5 μg/mL) for 3 h and subjected to immunofluorescence with an anti-GM130 antibody. Representative fluorescent microscopic images indicate colocalization of GalT (red) and *cis*-Golgi GM130 (white) in YFP-ArfGAP1-positive cells (green), whereas GalT signal often extends beyond the perinuclear GM130-positive Golgi complex. Presence (arrowhead) or absence (arrow) of GalT-positive Golgi-derived vesicles are observed in YFP-ArfGAP1 overexpressing cells. Scale bar: 25 μm. Graph indicates percent of ArfGAP1-positive cells containing GalT-positive vesicles (dispersed outside of the Golgi complex) following tunicamycin (or DMSO) treatment. Bars represent the mean ± SEM percent of cells displaying GalT-positive vesicles (*n* ≥ 3 independent experiments). **P < 0.01 or ****P < 0.0001 by two-way ANOVA with Tukey’s *post-hoc* analysis, as indicated. *ns*, non-significant.

As ArfGAP1 is involved in Golgi-to-ER retrograde transport (Donaldson and Jackson, 2011), we asked whether the altered localization of ArfGAP1 due to phosphorylation can influence the cellular response and Golgi sorting induced by ER stress. As such, SH-SY5Y cells transiently co-expressing YFP-ArfGAP1 variants (WT, 3A or 3D) and mCherry-N1-galactosyltransferase (GalT), a Golgi-resident enzyme, were treated with or without tunicamycin (2.5 μg/mL) for 3 h. Under basal conditions (DMSO treatment), the small percentage of cells displaying GalT-positive Golgi-derived vesicles are similar between control (empty vector) or WT ArfGAP1 conditions (Figure 9). ArfGAP1-3A expression markedly increases the number of cells with GalT-positive vesicles localized outside the Golgi complex (∼20%), whereas ArfGAP1-3D has an intermediate, yet non-significant effect compared to WT ArfGAP1 (Figure 9). Interestingly, while tunicamycin treatment that induces mild ER stress has no effect on the percent of cells with GalT-positive vesicles in the absence of ArfGAP1, the expression of WT or 3A ArfGAP1 produces a marked and equivalent increase in cells displaying GalT-positive vesicles (40–50%) (Figure 9). In contrast, ArfGAP1-3D has a small effect on the percent of cells with GalT-positive vesicles following tunicamycin treatment, similar to its effects in DMSO-treated cells (Figure 9). The mild ER stress induced by tunicamycin treatment did not qualitatively alter the number of cells in these assays. Our data indicate that ArfGAP1 expression robustly induces the formation of GalT-positive Golgi-derived vesicles in response to mild ER stress, consistent with an increase in Golgi-to-ER retrograde transport, whereas the phospho-mimic 3D mutant completely inhibits the normal activity of ArfGAP1 in this assay. These data are consistent with the altered cytoplasmic localization of ArfGAP1-3D compared to the WT and 3A proteins (Figure 8).

## Discussion

In this study we have evaluated the LRRK2-ArfGAP1 interaction, with a focus on the impact of candidate *in vitro* LRRK2-specific phosphorylation sites on ArfGAP1 localization and activity in cells. LRRK2 robustly and directly phosphorylates ArfGAP1 *in vitro*, with the most prominent phosphorylation sites residing within C-terminal residues 252–359 of ArfGAP1. Mass spectrometry analysis identified four phosphorylated threonine residues (pThr145, pThr189, pThr230, pThr292) in full-length ArfGAP1, with only one of these sites (pT292) localized within residues 252–359. A prior study identified additional LRRK2-dependent phosphorylation sites, including Ser284 within this region (Xiong et al., 2012), that were also incorporated into our study together with a known phosphorylation site at T291. Combining null mutations at these three sites (S284A, T291A, T292A) is sufficient to block LRRK2-mediated phosphorylation within residues 252–359 of ArfGAP1 *in vitro*. Using phospho-null (Ala, A) or phospho-mimic (Asp, D) substitutions combined at these three sites, we find that 3A and 3D mutations both impair ArfGAP1-induced Golgi complex fragmentation in human neural cells. We further find that blocking phosphorylation at these sites (3A mutant) is sufficient to attenuate neurite outgrowth deficits normally induced by ArfGAP1 in cultured primary neurons and rescues the pathogenic effects of G2019S LRRK2 on neurite outgrowth. We identify novel interacting proteins for ArfGAP1 in human neural cells, including mitochondrial proteins, and demonstrate that the phospho-mimic 3D mutation promotes the interaction of ArfGAP1 with mitochondrial VDACs. The enhanced interaction with VDACs most likely results from the increased cytoplasmic localization of the ArfGAP1-3D mutant and its impaired localization to the Golgi complex. Consistent with this altered localization, the 3D mutant completely blocks the ArfGAP1-induced formation of Golgi-derived vesicles following mild ER stress. Collectively, our study reveals the functional impact of selected candidate *in vitro* LRRK2-specific phosphorylation sites within ArfGAP1 and identifies the opposing actions of ArfGAP1 phosphorylation in i) maintaining Golgi localization and promoting Golgi complex dispersal (impaired by the phospho-mimic mutant) and in ii) mediating neurite outgrowth deficits (rescued by the phospho-null mutant). Our findings indicate that ArfGAP1 subcellular localization, protein interactions and activity are dynamically regulated by LRRK2-specific phosphorylation at these three sites, suggesting that the interaction between LRRK2 and ArfGAP1 is complex and non-linear with phosphorylation serving to regulate different aspects of ArfGAP1 function.

Our prior studies with ArfGAP1 demonstrated that its expression was critical for LRRK2-induced cellular toxicity in yeast or primary neuronal models (Stafa et al., 2012; Xiong et al., 2010), implying that ArfGAP1 normally plays a role in mediating the downstream pathogenic effects of mutant LRRK2. However, it was unclear whether such an effect results from the GAP-like activity of ArfGAP1 that enhances the GTP hydrolysis activity of LRRK2 (and other small GTPases), or whether it results from the direct phosphorylation of ArfGAP1 by LRRK2 and its downstream consequences on Golgi vesicle trafficking pathways (Stafa et al., 2012; Xiong et al., 2012). As a potential mechanism for explaining LRRK2-induced neurotoxicity, we have explored the role of ArfGAP1 as a LRRK2 substrate. A prior study suggested that mutation of six distinct serine or threonine residues located throughout the C-terminal half of ArfGAP1 completely prevented its phosphorylation by LRRK2, but the relative contribution of phosphorylation at each site was not clearly determined (Xiong et al., 2012). Here, by combining domain mapping and mutational studies, we have highlighted residues 252–359 as a major region for ArfGAP1 phosphorylation by LRRK2 and nominated three candidate phosphorylation sites in this region (S284, T291, and T292). These phosphorylation sites are located within a highly conserved lipid-packing sensor motif (ALPS2, residues 264–295) that is known to regulate the binding of ArfGAP1 to curved membranes in cooperation with the ALPS1 motif (Bigay et al., 2005; Mesmin et al., 2007). Notably, we also identified another phosphorylation site (pT230) within the ALPS1 motif (residues 199–234), not further studied here, suggesting a more general role for LRRK2 in regulating ArfGAP1 membrane association. Consistent with such a function, phospho-mimicking mutations at the ALPS2 sites (S284D/T291D/T292D) induced the relocalization of ArfGAP1 from the *cis*-Golgi compartment to the cytoplasm, where it exhibits an enhanced interaction with VDAC proteins at the mitochondrial outer membrane. The 3D mutation also impaired ArfGAP1-induced Golgi fragmentation and impaired the ER stress-induced production of Golgi-derived vesicles, all consistent with a diminished association of ArfGAP1-3D with the *cis*-Golgi membrane. Prior studies have shown that phosphorylation within the C-terminal domain re-localizes ArfGAP1 from the Golgi to the cytosol (Yu and Roth, 2002). Ser284, Thr291, and Thr292 are located on the polar face of the ALPS2 motif, rather than within the nonpolar hydrophobic face that directly interacts with membranes. Despite this, substitution of a polar uncharged side chain (Ser or Thr, -CH_2_OH or -CH_2_(OH)CH_3_) with a polar negatively charged (Asp, -CH_2_COOH) side chain mimicking phosphorylation is still sufficient to disrupt Golgi localization and fragmentation, implying the 3D mutant likely repulses membranes. Therefore, we suggest that LRRK2-mediated phosphorylation within the ALPS2 motif diminishes its affinity or specificity for highly curved membranes such as Golgi-derived vesicles. A parallel role has been ascribed to the LRRK2-mediated phosphorylation of endophilin-A in endocytosis, dynamically affecting its membrane association and tubulation via Ser75 phosphorylation within its membrane-sensing BAR domain (Matta et al., 2012). The ArfGAP1-3A mutant also impairs ArfGAP1-induced Golgi fragmentation yet adopts a normal subcellular localization at the *cis*-Golgi compartment. It is unclear whether these effects result from altered membrane affinity or dynamics, or perhaps from altered GAP activity directed at Golgi-localized small GTPases such as Arf1. While we have not directly tested the impact of ALPS2 motif phosphorylation on GAP activity, especially since the GAP domain is distant and oppositely resides in the N-terminal region of ArfGAP1, a prior study demonstrated that LRRK2-mediated phosphorylation impaired ArfGAP1-stimulated Arf1 GTP hydrolysis *in vitro* (Xiong et al., 2012). However, a non-phosphorylatable 6A mutant of ArfGAP1 retained the capacity to stimulate Arf1 GTPase activity *in vitro*, implying that it was fully functional (Xiong et al., 2012). Accordingly, in cells, we propose that ArfGAP1-3A does not impact its GAP activity but instead exerts its effects by altered membrane binding. Substitution of a hydrophobic side chain (Ala, -CH_3_) for polar side chains at these three sites in the polar face of ALPS2 could similarly alter membrane binding dynamics, yet without impacting the specificity for Golgi-derived vesicles. Prior studies have focused on the nonpolar face of the ALPS2 motif by substituting hydrophobic for negatively charged sides chains that repulse membranes and likely have a more disruptive effect on the formation of an amphipathic α-helical structure (Bigay et al., 2005; Mesmin et al., 2007). It is intriguing to consider that charge alterations of the Ser/Thr-rich polar face of ALPS2, such as via phosphorylation, can impact the membrane binding and function of ArfGAP1.

ALPS motifs play a central role in sensing membrane curvature, and target proteins onto these membranes depending on the degree of curvature (Bigay et al., 2005; Yu and Roth, 2002). ALPS are amphipathic helices where the hydrophobic-face residues adsorb to membranes that display lipid packing defects, and with a polar face enriched in serine and threonine residues (Bigay et al., 2005; Mesmin et al., 2007). ArfGAP1 contains two ALPS motifs (Mesmin et al., 2007). Interestingly, these ALPS motifs present differences regarding membrane curvature and lipid composition to which they bind, with ALPS1 binding to highly curved membranes whereas ALPS2 appears to tune this detection toward larger radii curved-membranes (Mesmin et al., 2007; Vanni et al., 2014). A previous study demonstrated that substitution of amino acids at the hydrophobic face of ALPS1 can modify ArfGAP1 cellular localization (Parnis et al., 2006). Here, we present evidence suggesting that the ArfGAP1-VDAC interaction could be regulated by LRRK2-dependent phosphorylation. This increased interaction does not imply that the ALPS2 motif interacts with VDAC, but rather we suggest that the shift between high and low curvature membrane detection induced by ALPS2 phosphorylation localizes ArfGAP1 to the outer mitochondrial membrane, thereby facilitating an ArfGAP1-VDAC interaction. Pathogenic LRRK2 mutants are associated with mitochondrial dysfunction (extensively reviewed in Singh et al., 2019), although it is debated whether LRRK2 affects mitochondria directly or indirectly. Previous studies have shown that cytosolic proteins can regulate VDAC function and affect mitochondrial outer membrane permeability (Maldonado et al., 2013; Rosencrans et al., 2021). Our data suggests that ArfGAP1 phosphorylation (induced by LRRK2) increases its interactions with VDAC and allows us to speculate that ArfGAP1 phosphorylation may play a pathogenic role in PD by regulating mitochondrial function. Additional experiments exploring the ArfGAP1-VDAC interaction will be required to support such a hypothesis.

Since ALPS motifs bind to membranes in a specific manner recognizing lipid packaging defects, it is interesting to consider ALPS phosphorylation as a functional regulatory mechanism for ArfGAP1 localization. Moreover, as the lipid composition of membranes varies between different organelles, it is plausible that ArfGAP1 phosphorylation by LRRK2 will modify its affinity for membranes with different curvature and/or lipid composition, thereby regulating ArfGAP1 localization and its role within different intracellular processes (Chorlay and Thiam, 2020). Such a mechanism for protein regulation has already been observed in yeast, where Vps41 can alternate between different trafficking routes depending on phosphorylation of its ALPS motif, suggesting phosphorylation could serve as a switch for Vps41 between endosome-to-vacuole and AP-3 vesicle-to-vacuole fusion (Cabrera et al., 2009; Cabrera et al., 2010; Ho and Stroupe, 2016). Adaptor proteins (AP) are key players in vesicular trafficking process between cellular compartments, connecting endosomes, Golgi, plasma membrane and lysosomes (Hirst et al., 2011; Rodriguez-Fernandez and Dell’Angelica, 2015). The interaction of ArfGAP1 with AP-2 and AP-3 has already been demonstrated, suggesting that ArfGAP1 is likely involved in other intracellular pathways in addition to retrograde Golgi-ER transport (Bai et al., 2011; Rodriguez-Fernandez and Dell’Angelica, 2015; Saha et al., 2021). Accordingly, Meng and colleagues recently showed that ArfGAP1 binds to mTORC1, inhibiting its activation and promoting autophagy activation (Meng et al., 2021). Together, ALPS phosphorylation is likely to influence ArfGAP1 localization and intracellular functions by regulating which proteins and membranes it can interact with.

The capacity of ArfGAP1 expression to modulate neurite outgrowth in cultured primary neurons suggests a potential role in regulating neuronal process complexity or integrity. However, there are limited studies on ArfGAP1 in the context of brain localization, structure or function, especially due to a paucity of specific antibodies (Chornyy et al., 2017; Parnis et al., 2006). Our prior study demonstrated that endogenous ArfGAP1 is detected in multiple brain regions and is localized within different primary neurons, and WT ArfGAP1 overexpression was sufficient to inhibit neurite outgrowth, similar to PD-linked LRRK2 mutants (Stafa et al., 2012). Here, we show that blocking LRRK2 phosphorylation via the 3A mutant impairs the capacity of ArfGAP1 to regulate neurite outgrowth. This might imply that ArfGAP1 phosphorylation *per se* is required for this activity, as suggested by the similar effects of WT and phospho-mimic 3D ArfGAP1. Consistent with this idea, we have previously shown that endogenous LRRK2 is required, at least in part, for the effects of ArfGAP1 on neurite outgrowth (Stafa et al., 2012). Our data also reveals that 3A ArfGAP1 is able to rescue in part the robust effects of G2019S LRRK2 on neurite outgrowth inhibition. The mechanism underlying this interesting rescue effect is unclear but it could be due to interference with the normal phosphorylation of endogenous ArfGAP1 perhaps via sequestration of, or excessive binding to, LRRK2. Supporting this idea, we have previously shown that reducing ArfGAP1 expression is sufficient to protect against G2019S LRRK2 in these neurite outgrowth assays (Stafa et al., 2012). It is therefore tempting to speculate that non-phosphorlyatable ArfGAP1 peptides could represent an interesting strategy for attenuating the pathogenic effects of mutant LRRK2 in future studies.

Collectively, the present study together with prior work (Stafa et al., 2012; Xiong et al., 2010; Xiong et al., 2012) provides further evidence for a functional interaction between LRRK2 and ArfGAP1 that serves to regulate ArfGAP1 subcellular localization, protein interactions, activity and neuronal integrity via LRRK2-mediated phosphorylation of its ALPS2 motif. Our data suggests a complex relationship between ArfGAP1 and LRRK2 in the pathogenesis of PD, linking Golgi-ER retrograde sorting and mitochondrial pathways through ArfGAP1 phosphorylation. Our findings support additional validation of ArfGAP1 as a candidate LRRK2 substrate and therapeutic target for modulating *LRRK2*-linked PD.

## Materials and methods

### Animals

Timed pregnant female Sprague-Dawley outbred rats were obtained from Taconic Biosciences and P1 rats were used to prepare post-natal primary cortical neuronal cultures as previously reported (Stafa et al., 2012). Rats were maintained in a pathogen-free barrier facility and provided with food and water *ad libitum* and exposed to a 12 h light/dark cycle. Animals were treated in strict accordance with the NIH Guidelines for the Care and Use of Laboratory Animals. All animal experiments were approved by the Van Andel Institute Institutional Animal Care and Use Committee (IACUC).

### Expression plasmids, proteins, and antibodies

Mammalian expression plasmids containing FLAG-tagged full length human LRRK2 (WT, R1441C and G2019S) variants were kindly provided by Dr. Christopher Ross (Johns Hopkins University, Baltimore, United States) (Smith et al., 2005). A plasmid encoding mCherry-N1-Galactosyltransferase (GalT) was obtained from Addgene (#87327) (Tie et al., 2016). A plasmid containing DsRed-Max-N1 was obtained from Addgene (#21718) (Strack et al., 2008). A C-terminal YFP-tagged rat ArfGAP1 plasmid was kindly provided by Dr. Jennifer Lippincott-Schwartz (National Institutes of Health, Bethesda, United States) (Liu et al., 2005). ArfGAP1 phospho-null/phospho-mimic mutations (S284A, S284D, T291A, T291D, T292A, T292D, S284A/T291A/T292A, and S284D/T291D/T292D) were generated by site-directed mutagenesis using the Stratagene QuickChange II XL kit (Agilent Technologies, La Jolla, CA, United States) and DNA sequencing was performed to confirm integrity.

Recombinant GST-tagged human LRRK2 proteins (residues 970–2,527) were obtained from Invitrogen (Carlsbad, CA, United States). GST-tagged rat ArfGAP1 plasmids (full-length or deletion mutants [residues 1–415, 1–136, 137–251, 252–359, 137–415, and 360–415] in pGEX-6P1) were kindly provided by Dr. Victor Hsu (Brigham and Women’s Hospital, Harvard Medical School) (Bai et al., 2011). Phospho-null variants (S284A, T291A, T292A, and S284A/T291A/T292A) were introduced into a 252–359 (F5) GST-ArfGAP1 plasmid by site-directed mutagenesis. GST-ArfGAP1 proteins were purified from IPTG-induced bacteria using Glutathione-Sepharose 4B columns (GST Bulk Kit, Cytiva) following standard protocols as described (Schäfer et al., 2015).

The following primary antibodies were employed: mouse anti-FLAG (clone M2; Sigma-Aldrich, Buchs, Switzerland), mouse anti-GFP (clones 7.1 and 13.1; Roche Applied Science, Basel, Switzerland), mouse anti-GM130 (clone 35; BD Biosciences), goat anti-GST-HRP (RPN1236, GE Healthcare), mouse anti-TOMM20 (ab56783, Abcam), mouse anti-PGK1 (459250, Invitrogen), rabbit anti-LAMP1 (ab24170, Abcam), rabbit anti-Lamin B1 (ab16048, Abcam), and rabbit anti-pan-VDAC1-3 (PA1-954A, Thermo Fisher). Secondary antibodies included: HRP-coupled anti-mouse and anti-rabbit IgG, light chain-specific (Jackson ImmunoResearch, Inc., West Grove, PA, United States), and anti-rabbit IgG and anti-mouse IgG coupled to AlexaFluor-488, -546 and -633 (Invitrogen).

### Cell culture, transfection, and treatment

Human SH-SY5Y neural cells were maintained at 37°C with 5% CO_2_ atmosphere in Dulbecco’s Modified Eagle’s Media (DMEM) (Gibco) supplemented with 10% (v/v) fetal bovine serum and penicillin/streptomycin. Cell transfection was performed using plasmid DNA and XtremeGene HP DNA Transfection reagent (Roche), according to manufacturer’s instructions. For endoplasmic reticulum stress assays, 48 h after co-transfection, cells were treated with tunicamycin (2.5 μg/mL) or DMSO for 3 h, followed by immunocytochemical analysis. Primary cortical neurons were maintained in 35 mm dishes on glass coverslips in Neurobasal media containing B27 supplement (2% w/v), L-glutamine (500 μM) and penicillin/streptomycin (100 U/mL) as previously described (Stafa et al., 2012).

### Immunocytochemistry

SH-SY5Y neural cells were transiently transfected with YFP-ArfGAP1 variants, or co-transfected with YFP-ArfGAP1 and mCherry-N1-Galactosyltransferase (GalT) (Addgene #87327) at a 3:1 molar ratio. After 48 h, medium was removed, and cells were fixed with 4% PFA at room temperature for 20 min and then washed 3 times with 1X PBS (pH 7.4). Non-specific binding was blocked by incubation in 5% BSA in PBS at room temperature. Cells were incubated overnight at 4°C with anti-TOMM20 (Abcam) or anti-GM130 (BD Biosciences) antibodies, followed by incubation with AlexaFluor-conjugated secondary antibodies at room temperature for 90 min. Coverslips were mounted onto glass slides using Prolong Diamond Antifade Mountant with DAPI (Thermo Fisher). Fluorescence images were acquired by microscopy using a Nikon A1plus-RSi scanning confocal microscope or a Leica DM5500B epifluorescence microscope.

### Subcellular fractionation

For subcellular fractionation, we followed the protocol from Abas and Luschnig (2010) with some modifications. Culture medium was removed, cells were carefully washed once with PBS and resuspended in extraction buffer (EB): 100 mM Tris-HCl pH 7.5, 25% (w/w) sucrose, 5% (v/v) glycerol, 10 mM EDTA, 5 mM KCl, supplemented with Complete Mini Protease inhibitor EDTA-free cocktail (Roche) and Phosphatase Inhibitor cocktails 2 and 3 (Sigma Aldrich). Cells were homogenized using a 25G needle at 4°C, cell lysis was monitored with trypan blue, and samples were centrifuged once lysis was > 95%. Briefly, lysates were centrifuged for 5 min at 800*g*. The nuclear fraction (pellet 1) was washed 2 times using EB. Supernatant was centrifuged for 20 min at 10,000 *g*, and the mitochondrial fraction (pellet 2) was washed 2 times with EB. Supernatant was centrifuged for 120 min at 21,100 *g*, the microsomal fraction (pellet 3) was washed twice with EB, and supernatant was saved as the cytosolic fraction. Samples were subjected to Western blot analysis.

### Co-immunoprecipitation

For co-immunoprecipitation (IP) assays, SH-SY5Y cells were transiently transfected with the desired amount of plasmid in 10 cm dishes. At 48 h post-transfection, media was removed, and cells were harvested in 1 mL of lysis buffer [1X PBS, 1% Triton X-100, 1X Complete Mini Protease inhibitor cocktail (Roche)]. Cell lysates were allowed to rotate 2 h at 4°C, and then centrifuged for 15 min at 15,000 rpm at 4°C. Supernatants were incubated overnight at 4°C with 50 μL Protein G-Dynabeads (Thermo Fisher) that had been pre-incubated for 2 h with anti-GFP antibody (2 μg; Roche). Samples were washed 3 times with 1X PBS, 1% Triton X-100 and once with PBS. IPs were eluted in 2X Laemmli buffer at 95°C for 5 min and analyzed by Western blot.

### Western blot analysis

Samples from IPs, input lysates, or subcellular fractionations were subjected to SDS-PAGE and transferred to nitrocellulose membranes (0.2 μm; GE Healthcare). Membranes were blocked for 1 h with 5% (w/v) nonfat milk and 0.1% Tween 20 in Tris-Buffered Saline (TBS-T) and incubated overnight at 4°C with primary antibodies: anti-FLAG, anti-GST-HRP, Anti-GM130, anti-VDAC, anti-GFP, anti-TOMM20, anti-PGK1, anti-LAMP1 or anti-Lamin B1. After primary antibody incubation, membranes were extensively washed with TBS-T, followed by 1 h incubation with HRP-conjugated secondary antibodies. Finally, membranes were washed and incubated with chemiluminescence reagents (ECL; GE Life Sciences) and visualized on a FujiFilm LAS-4000 Image Analysis system.

### *In vitro* kinase assays

In total, 300 ng of purified recombinant GST-tagged rat ArfGAP1 proteins (F5 252-359 fragment: WT, S284A, T291A or T292A) were incubated with 95 ng of recombinant GST-tagged human LRRK2 (residues 970–2,527: G2019S or D1994A; Invitrogen) in 5 μL 10x kinase buffer (Cell Signaling Technology) and 2 μL [^33^P]-γ-ATP (0.2 μCi/reaction) in a final volume of 15 μL by shaking at 30°C for 30 min. Where indicated, an increasing concentration of F5 GST-ArfGAP1 protein (WT or 3A mutant; 75, 300, 500 or 750 ng) was added with LRRK2 (G2019S or D1994A) in the samples and incubated for 30 min in similar fashion. The reaction was terminated by adding 5 μL of 4x LDS sample buffer (Invitrogen) followed by denaturing the samples at 70°C for 10 min. Samples were resolved by SDS-PAGE, transferred onto PVDF membranes (GE Life Sciences) and subjected to autoradiographic detection using a Typhoon Phosphorimager (Cytiva) as described previously (Nguyen et al., 2018). Membranes were subsequently blocked with TBS-T containing 5% milk and incubated with anti-GST-HRP antibody (GE Life Sciences). Proteins were visualized using enhanced chemiluminescence (GE Life Sciences) and a luminescent image analyzer (LAS-4000, FujiFilm). Similar kinase assays were conducted using recombinant full-length GST-ArfGAP1 and GST-ΔN-LRRK2 (WT, G2019S or D1994A) in the presence of excess cold ATP, for analysis by mass spectrometry. In separate radioactive kinase assays with [^33^P]-γ-ATP, we incubated recombinant GST-tagged ArfGAP1 deletion mutants (WT or F1-F5) with purified full-length FLAG-tagged LRRK2 (WT, G2019S or D1994A) derived by anti-FLAG IP from transfected SH-SY5Y cells, as described (Stafa et al., 2014). Membranes were first imaged for ^33^P by autoradiography using a Typhoon Phosphorimager, and then by Western blotting with anti-FLAG-HRP and anti-GST-HRP antibodies.

### Cortical neurite length assay

Rat primary cortical cultures were co-transfected at DIV 3 with FLAG-LRRK2 and DsRed-Max-N1 at a 10:1 molar ratio using Lipofectamine 2000 reagent (Invitrogen) according to manufacturer’s recommendations. For co-expression experiments, transfections were performed with FLAG-LRRK2, YFP-ArfGAP1 (WT, phospho-null / phospho-mimic mutants) and DsRed plasmids at a 10:10:1 molar ratio. At DIV 6, cultures were fixed with 4% paraformaldehyde and processed for immunocytochemistry with mouse anti-FLAG (M2) antibody (Sigma-Aldrich) and anti-mouse IgG-AlexaFluor-488 or -633 antibodies (Invitrogen). Fluorescent images were acquired, processed and analyzed for neurite length measurement similar to our previous study (Stafa et al., 2012).

### Golgi fragmentation assay

SH-SY5Y cells were transiently transfected with YFP-ArfGAP1 (WT, phospho-null / phospho-mimic mutants), fixed and processed for immunocytochemistry with mouse anti-GM130 antibody and anti-mouse-IgG AlexaFluor-546. Golgi morphology was assessed by classifying complexes as either normal intact, partially fragmented, or fully fragmented, as previously reported (Stafa et al., 2012).

### Mass spectrometry

We performed similar *in vitro* kinase assays with excess non-radioactive ATP and measured the phosphorylation of recombinant GST-tagged WT ArfGAP1 by GST-ΔN-LRRK2 (WT, G2019S and D1994A) using mass spectrometry. An in-solution digestion protocol was performed as detailed previously (Nolte et al., 2015). Briefly, samples were lysed, and proteins were reduced with dithiothreitol (DTT) followed by alkylation with iodoacetamide (IAA) in the dark. Protein digestion was performed with trypsin (enzyme:substrate ratio of 1:100) (Wako) at room temperature for 3 h followed by an additional overnight digestion with Lys-C. The digestion was stopped by acidification and the mixture was desalted on reversed phase C18 StageTips prior to liquid chromatography tandem mass spectrometry (LC-MS/MS, Thermo Scientific Q-Exactive HF-X; Michigan State University Proteomics Facility, East Lansing, MI). In separate experiments, LC-MS/MS analysis was conducted to detect ArfGAP1 protein interactors in anti-GFP IP samples derived from SH-SY5Y cells transiently expressing YFP-tagged WT ArfGAP1 versus an empty plasmid control.

### Raw data processing

The mass spectrometric raw data were processed and analyzed using MaxQuant software (version 1.4.7.2) (Cox and Mann, 2008). Proteins were detected using the implemented Andromeda search engine and human/rat database with common contaminants. Oxidation of methionine and acetylation of the protein N-terminus were selected for variable modifications, whereas carbamidomethylation of cysteines were chosen for fixed modification. Lys-C was taken as the protease where a maximum of 2 missed cleavages were allowed. For fragment ions, 0.02 Da mass tolerances were selected and for identification, only peptides with a minimum of six amino acids were considered. The false discovery rate was set to 1% on the peptide-spectrum-match and protein level using the implemented decoy algorithm. The minimum ratio count was set to 2. Data analysis and visualization were performed using Perseus software (Max Plank Institute, Martinsried) and the statistical environment R. Significant differentially-expressed phosphoproteins or proteins were identified by a permutation-based FDR approach using a cutoff of 0.01 and 500 permutations. Moreover, significant differentially expressed interacting proteins were identified by taking log2 fold change ≥ 2. Interaction networks, GO, KEGG, and INTERPRO domain analysis were done by STRING (Szklarczyk et al., 2015), the statistical environment R and Perseus (Tyanova et al., 2016).

### Statistical analysis

All data were analyzed by two-tailed, unpaired Student’s *t*-test for pair-wise comparisons, one-way ANOVA with Dunnett’s multiple comparisons test for samples grouped by one factor, or two-way ANOVA with Tukey’s multiple comparisons test for samples grouped by two factors, as indicated. *P* < 0.05 was considered significant.

## Data Availability

The original contributions presented in the study are included in the article/Supplementary material, further inquiries can be directed to the corresponding author.

## Supplementary material

Supplementary Figure S1 Impact of single phosphorylation mutants on ArfGAP1-induced Golgi fragmentation in neural cells. **(A)** SH-SY5Y cells transiently expressing YFP-tagged WT ArfGAP1 or empty vector were fixed and subjected to immunofluorescence with antibodies to different Golgi membrane markers (GM130, TGN46, Giantin, GOLGA4). Quantitation of Golgi morphology in ArfGAP1-positive cells reveals similar levels of Golgi fragmentation between Golgi markers (90–100% cells). **(B,C)** Quantitation of Golgi fragmentation induced by overexpression of WT or single phospho-mutants of YFP-ArfGAP1 in SH-SY5Y cells. Single phospho-null or phospho-mimic mutants induce similar Golgi fragmentation levels to WT ArfGAP1 (∼85% cells). **(D)** Golgi fragmentation induced by ArfGAP1 double (T291A/T292A) or triple (3A) phospho-null mutants indicates partial Golgi fragmentation of double mutants (∼65% cells) or 3A mutant (∼45% cells) compared to WT ArfGAP1 (∼85% cells). In each graph, bars represent the percentage of cells with normal or fragmented Golgi from a representative experiment.

Supplementary Figure S2 Impact of single phospho-null mutants on ArfGAP1-induced inhibition of neurite outgrowth. **(A)** Rat primary cortical neurons were co-transfected at DIV3 with YFP-ArfGAP1 (WT, S284A, T291A or T292A) or empty vector and DsRed-Max-N1 plasmids, and fixed at DIV6 for confocal fluorescence microscopy analysis. Fluorescent images reveal the co-labeling of cortical neurons with YFP-ArfGAP1 (green) and DsRed (red), with the DsRed images pseudo colored (ICA) to enhance the contrast of neuritic processes. Neuronal soma (white arrows) and axonal processes (black arrowheads) are indicated. Scale bars: 50 μm. **(B)** Quantitative analysis of DsRed-positive axon length in YFP-ArfGAP1-positive neurons or control neurons (empty vector) is shown. Bars represent the mean ± SEM axon length (in μm) from 90 to 120 double DsRed-/YFP-ArfGAP1-positive neurons, or single DsRed-positive neurons (control), across three independent experiments/cultures (*n* = 3). *****P* < 0.0001 as indicated compared to ArfGAP1-WT by one-way ANOVA with Tukey’s multiple comparisons test. *ns*, non-significant.

Supplementary Figure S3 Impact of single phospho-mimic mutants on ArfGAP1-induced inhibition of neurite outgrowth. **(A)** Rat primary cortical neurons were co-transfected at DIV3 with YFP-ArfGAP1 (WT, S284D, T291D or T292D) or empty vector and DsRed-Max-N1 plasmids, and fixed at DIV6 for confocal fluorescence microscopy analysis. Fluorescent images reveal the co-labeling of cortical neurons with YFP-ArfGAP1 (green) and DsRed (red), with the DsRed images pseudo colored (ICA) to enhance the contrast of neuritic processes. Neuronal soma (white arrows) and axonal processes (black arrowheads) are indicated. Scale bars: 50 μm. **(B)** Quantitative analysis of DsRed-positive axon length in YFP-ArfGAP1-positive neurons or control neurons (empty vector) is shown. Bars represent the mean ± SEM axon length (in μm) from 90 to 120 double DsRed-/YFP-ArfGAP1-positive neurons, or single DsRed-positive neurons (control), across three independent experiments/cultures (*n* = 3). *****P* < 0.0001 as indicated compared to ArfGAP1-WT by one-way ANOVA with Tukey’s multiple comparisons test. *ns*, non-significant.

## References

[B1] Aasly J. O. Vilariño-Güell C. Dachsel J. C. Webber P. J. West A. B. Haugarvoll K. et al. (2010). Novel pathogenic LRRK2 p.Asn1437His substitution in familial Parkinson’s disease. *Mov. Disord.* 25 2156–2163. 10.1002/mds.23265 20669305 PMC2970614

[B2] Abas L. Luschnig C. (2010). Maximum yields of microsomal-type membranes from small amounts of plant material without requiring ultracentrifugation. *Anal. Biochem.* 401 217–227. 10.1016/j.ab.2010.02.030 20193653 PMC3685806

[B3] Bai M. Gad H. Turacchio G. Cocucci E. Yang J. S. Li J. et al. (2011). ARFGAP1 promotes AP-2-dependent endocytosis. *Nat. Cell Biol.* 13 559–567. 10.1038/ncb2221 21499258 PMC3087831

[B4] Belluzzi E. Gonnelli A. Cirnaru M. D. Marte A. Plotegher N. Russo I. et al. (2016). LRRK2 phosphorylates pre-synaptic N-ethylmaleimide sensitive fusion (NSF) protein enhancing its ATPase activity and SNARE complex disassembling rate. *Mol. Neurodegener.* 11:1. 10.1186/s13024-015-0066-z 26758690 PMC4711005

[B5] Bigay J. Casella J. F. Drin G. Mesmin B. Antonny B. (2005). ArfGAP1 responds to membrane curvature through the folding of a lipid packing sensor motif. *EMBO J.* 24 2244–2253. 10.1038/sj.emboj.7600714 15944734 PMC1173154

[B6] Biosa A. Trancikova A. Civiero L. Glauser L. Bubacco L. Greggio E. et al. (2013). GTPase activity regulates kinase activity and cellular phenotypes of Parkinson’s disease-associated LRRK2. *Hum. Mol. Genet.* 22 1140–1156. 10.1093/hmg/dds522 23241358

[B7] Biskup S. West A. B. (2009). Zeroing in on LRRK2-linked pathogenic mechanisms in Parkinson’s disease. *Biochim. Biophys. Acta* 1792 625–633. 10.1016/j.bbadis.2008.09.015 18973807 PMC2745057

[B8] Blauwendraat C. Nalls M. A. Singleton A. B. (2020). The genetic architecture of Parkinson’s disease. *Lancet Neurol.* 19 170–178. 10.1016/S1474-4422(19)30287-X 31521533 PMC8972299

[B9] Cabrera M. Langemeyer L. Mari M. Rethmeier R. Orban I. Perz A. et al. (2010). Phosphorylation of a membrane curvature-sensing motif switches function of the HOPS subunit Vps41 in membrane tethering. *J. Cell Biol.* 191 845–859. 10.1083/jcb.201004092 21079247 PMC2983053

[B10] Cabrera M. Ostrowicz C. W. Mari M. LaGrassa T. J. Reggiori F. Ungermann C. (2009). Vps41 phosphorylation and the Rab Ypt7 control the targeting of the HOPS complex to endosome-vacuole fusion sites. *Mol. Biol. Cell* 20 1937–1948. 10.1091/mbc.e08-09-0943 19193765 PMC2663929

[B11] Chang D. Nalls M. A. Hallgrímsdóttir I. B. Hunkapiller J. van der Brug M. Cai F. et al. (2017). A meta-analysis of genome-wide association studies identifies 17 new Parkinson’s disease risk loci. *Nat. Genet.* 49 1511–1516. 10.1038/ng.3955 28892059 PMC5812477

[B12] Chorlay A. Thiam A. R. (2020). Neutral lipids regulate amphipathic helix affinity for model lipid droplets. *J. Cell Biol.* 219:e201907099. 10.1083/jcb.201907099 32328636 PMC7147095

[B13] Chornyy S. Parnis A. Shmoish M. Cassel D. (2017). High abundance of ArfGAP1 found in the mossy fibers in hilus of the dentate gyrus region of the mouse brain. *PLoS One* 12:e0189659. 10.1371/journal.pone.0189659 29240824 PMC5730162

[B14] Cookson M. R. (2015). LRRK2 pathways leading to neurodegeneration. *Curr. Neurol. Neurosci. Rep.* 15:42. 10.1007/s11910-015-0564-y 26008812 PMC5839465

[B15] Cox J. Mann M. (2008). MaxQuant enables high peptide identification rates, individualized p.p.b.-range mass accuracies and proteome-wide protein quantification. *Nat. Biotechnol.* 26 1367–1372. 10.1038/nbt.1511 19029910

[B16] Cukierman E. Huber I. Rotman M. Cassel D. (1995). The ARF1 GTPase-activating protein: Zinc finger motif and Golgi complex localization. *Science* 270 1999–2002. 10.1126/science.270.5244.1999 8533093

[B17] Deyaert E. Wauters L. Guaitoli G. Konijnenberg A. Leemans M. Terheyden S. et al. (2017). A homologue of the Parkinson’s disease-associated protein LRRK2 undergoes a monomer-dimer transition during GTP turnover. *Nat. Commun.* 8:1008. 10.1038/s41467-017-01103-4 29044096 PMC5714945

[B18] Donaldson J. G. Cassel D. Kahn R. A. Klausner R. D. (1992). ADP-ribosylation factor, a small GTP-binding protein, is required for binding of the coatomer protein beta-COP to Golgi membranes. *Proc. Natl. Acad. Sci. U. S. A.* 89 6408–6412. 10.1073/pnas.89.14.6408 1631136 PMC49510

[B19] Donaldson J. G. Jackson C. L. (2011). ARF family G proteins and their regulators: Roles in membrane transport, development and disease. *Nat. Rev. Mol. Cell Biol.* 12 362–375. 10.1038/nrm3117 21587297 PMC3245550

[B20] Dusonchet J. Li H. Guillily M. Liu M. Stafa K. Derada Troletti C. et al. (2014). A Parkinson’s disease gene regulatory network identifies the signaling protein RGS2 as a modulator of LRRK2 activity and neuronal toxicity. *Hum. Mol. Genet.* 23 4887–4905. 10.1093/hmg/ddu202 24794857 PMC4140468

[B21] Eugster A. Frigerio G. Dale M. Duden R. (2000). COP I domains required for coatomer integrity, and novel interactions with ARF and ARF-GAP. *EMBO J.* 19 3905–3917. 10.1093/emboj/19.15.3905 10921873 PMC306616

[B22] Gillardon F. (2009). Leucine-rich repeat kinase 2 phosphorylates brain tubulin-beta isoforms and modulates microtubule stability–a point of convergence in parkinsonian neurodegeneration? *J. Neurochem.* 110 1514–1522. 10.1111/j.1471-4159.2009.06235.x 19545277

[B23] Greggio E. Jain S. Kingsbury A. Bandopadhyay R. Lewis P. Kaganovich A. et al. (2006). Kinase activity is required for the toxic effects of mutant LRRK2/dardarin. *Neurobiol. Dis.* 23 329–341. 10.1016/j.nbd.2006.04.001 16750377

[B24] Healy D. G. Falchi M. O’Sullivan S. S. Bonifati V. Durr A. Bressman S. et al. (2008). Phenotype, genotype, and worldwide genetic penetrance of LRRK2-associated Parkinson’s disease: A case-control study. *Lancet Neurol.* 7 583–590. 10.1016/S1474-4422(08)70117-0 18539534 PMC2832754

[B25] Hirst J. Barlow L. D. Francisco G. C. Sahlender D. A. Seaman M. N. Dacks J. B. et al. (2011). The fifth adaptor protein complex. *PLoS Biol.* 9:e1001170. 10.1371/journal.pbio.1001170 22022230 PMC3191125

[B26] Ho R. Stroupe C. (2016). The HOPS/Class C Vps complex tethers high-curvature membranes via a direct protein-membrane interaction. *Traffic* 17 1078–1090. 10.1111/tra.12421 27307091

[B27] Islam M. S. Moore D. J. (2017). Mechanisms of LRRK2-dependent neurodegeneration: Role of enzymatic activity and protein aggregation. *Biochem. Soc. Trans.* 45 163–172. 10.1042/BST20160264 28202670 PMC5521802

[B28] Islam M. S. Nolte H. Jacob W. Ziegler A. B. Pütz S. Grosjean Y. et al. (2016). Human R1441C LRRK2 regulates the synaptic vesicle proteome and phosphoproteome in a *Drosophila* model of Parkinson’s disease. *Hum. Mol. Genet.* 25 5365–5382. 10.1093/hmg/ddw352 27794539 PMC6078604

[B29] Kalia L. V. Kalia S. K. Lang A. E. (2015). Disease-modifying strategies for Parkinson’s disease. *Mov. Disord.* 30 1442–1450. 10.1002/mds.26354 26208210

[B30] Kanao T. Venderova K. Park D. S. Unterman T. Lu B. Imai Y. (2010). Activation of FoxO by LRRK2 induces expression of proapoptotic proteins and alters survival of postmitotic dopaminergic neuron in *Drosophila*. *Hum. Mol. Genet.* 19 3747–3758. 10.1093/hmg/ddq289 20624856

[B31] Klausner R. D. Donaldson J. G. Lippincott-Schwartz J. (1992). Brefeldin a: Insights into the control of membrane traffic and organelle structure. *J. Cell Biol.* 116 1071–1080. 10.1083/jcb.116.5.1071 1740466 PMC2289364

[B32] Krumova P. Reyniers L. Meyer M. Lobbestael E. Stauffer D. Gerrits B. et al. (2015). Chemical genetic approach identifies microtubule affinity-regulating kinase 1 as a leucine-rich repeat kinase 2 substrate. *FASEB J.* 29 2980–2992. 10.1096/fj.14-262329 25854701

[B33] Lee S. Liu H. P. Lin W. Y. Guo H. Lu B. (2010). LRRK2 kinase regulates synaptic morphology through distinct substrates at the presynaptic and postsynaptic compartments of the *Drosophila* neuromuscular junction. *J. Neurosci.* 30 16959–16969. 10.1523/JNEUROSCI.1807-10.2010 21159966 PMC3045823

[B34] Liu W. Duden R. Phair R. D. Lippincott-Schwartz J. (2005). ArfGAP1 dynamics and its role in COPI coat assembly on Golgi membranes of living cells. *J. Cell Biol.* 168 1053–1063. 10.1083/jcb.200410142 15795316 PMC2171832

[B35] Maldonado E. N. Sheldon K. L. DeHart D. N. Patnaik J. Manevich Y. Townsend D. M. et al. (2013). Voltage-dependent anion channels modulate mitochondrial metabolism in cancer cells: Regulation by free tubulin and erastin. *J. Biol. Chem.* 288 11920–11929. 10.1074/jbc.M112.433847 23471966 PMC3636879

[B36] Martin I. Kim J. W. Lee B. D. Kang H. C. Xu J. C. Jia H. et al. (2014). Ribosomal protein s15 phosphorylation mediates LRRK2 neurodegeneration in Parkinson’s disease. *Cell* 157 472–485. 10.1016/j.cell.2014.01.064 24725412 PMC4040530

[B37] Matta S. Van Kolen K. da Cunha R. van den Bogaart G. Mandemakers W. Miskiewicz K. et al. (2012). LRRK2 controls an EndoA phosphorylation cycle in synaptic endocytosis. *Neuron* 75 1008–1021. 10.1016/j.neuron.2012.08.022 22998870

[B38] Meng D. Yang Q. Melick C. H. Park B. C. Hsieh T. S. Curukovic A. et al. (2021). ArfGAP1 inhibits mTORC1 lysosomal localization and activation. *EMBO J.* 40:e106412. 10.15252/embj.2020106412 33988249 PMC8204869

[B39] Mesmin B. Drin G. Levi S. Rawet M. Cassel D. Bigay J. et al. (2007). Two lipid-packing sensor motifs contribute to the sensitivity of ArfGAP1 to membrane curvature. *Biochemistry* 46 1779–1790. 10.1021/bi062288w 17253781

[B40] Myasnikov A. Zhu H. Hixson P. Xie B. Yu K. Pitre A. et al. (2021). Structural analysis of the full-length human LRRK2. *Cell* 184 3519–3527.e10. 10.1016/j.cell.2021.05.004 34107286 PMC8887629

[B41] Nalls M. A. Pankratz N. Lill C. M. Do C. B. Hernandez D. G. Saad M. et al. (2014). Large-scale meta-analysis of genome-wide association data identifies six new risk loci for Parkinson’s disease. *Nat. Genet.* 46 989–993. 10.1038/ng.3043 25064009 PMC4146673

[B42] Nguyen A. P. T. Daniel G. Valdés P. Islam M. S. Schneider B. L. Moore D. J. (2018). G2019S LRRK2 enhances the neuronal transmission of tau in the mouse brain. *Hum. Mol. Genet.* 27 120–134. 10.1093/hmg/ddx389 29088368

[B43] Nguyen M. Krainc D. (2018). LRRK2 phosphorylation of auxilin mediates synaptic defects in dopaminergic neurons from patients with Parkinson’s disease. *Proc. Natl. Acad. Sci. U. S. A.* 115 5576–5581. 10.1073/pnas.1717590115 29735704 PMC6003526

[B44] Nolte H. Hölper S. Housley M. P. Islam S. Piller T. Konzer A. et al. (2015). Dynamics of zebrafish fin regeneration using a pulsed SILAC approach. *Proteomics* 15 739–751. 10.1002/pmic.201400316 25504979

[B45] Obeso J. A. Stamelou M. Goetz C. G. Poewe W. Lang A. E. Weintraub D. et al. (2017). Past, present, and future of Parkinson’s disease: A special essay on the 200th Anniversary of the Shaking Palsy. *Mov. Disord.* 32 1264–1310. 10.1002/mds.27115 28887905 PMC5685546

[B46] Paisán-Ruíz C. Jain S. Evans E. W. Gilks W. P. Simón J. van der Brug M. et al. (2004). Cloning of the gene containing mutations that cause PARK8-linked Parkinson’s disease. *Neuron* 44 595–600. 10.1016/j.neuron.2004.10.023 15541308

[B47] Parnis A. Rawet M. Regev L. Barkan B. Rotman M. Gaitner M. et al. (2006). Golgi localization determinants in ArfGAP1 and in new tissue-specific ArfGAP1 isoforms. *J. Biol. Chem.* 281 3785–3792. 10.1074/jbc.M508959200 16316994

[B48] Peters P. J. Hsu V. W. Ooi C. E. Finazzi D. Teal S. B. Oorschot V. et al. (1995). Overexpression of wild-type and mutant ARF1 and ARF6: Distinct perturbations of nonoverlapping membrane compartments. *J. Cell Biol.* 128 1003–1017. 10.1083/jcb.128.6.1003 7896867 PMC2120412

[B49] Pevzner I. Strating J. Lifshitz L. Parnis A. Glaser F. Herrmann A. et al. (2012). Distinct role of subcomplexes of the COPI coat in the regulation of ArfGAP2 activity. *Traffic* 13 849–856. 10.1111/j.1600-0854.2012.01349.x 22375848

[B50] PfefferS. R. AlessiD. R. (2025). Leucine-rich repeat kinase 2: Pathways to Parkinson’s disease. *Cold Spring Harb. Perspect. Med.* 10.1101/cshperspect.a041620 [Epub ahead of print]. 40588344

[B51] Ponnalagu D. Singh H. (2017). Anion channels of mitochondria. *Handb. Exp. Pharmacol.* 240 71–101. 10.1007/164_2016_39 27783269 PMC5855116

[B52] Rawet M. Levi-Tal S. Szafer-Glusman E. Parnis A. Cassel D. (2010). ArfGAP1 interacts with coat proteins through tryptophan-based motifs. *Biochem. Biophys. Res. Commun.* 394 553–557. 10.1016/j.bbrc.2010.03.017 20211604

[B53] Rodriguez-Fernandez I. A. Dell’Angelica E. C. (2015). Identification of *Atg2* and *ArfGAP1* as candidate genetic modifiers of the eye pigmentation phenotype of adaptor protein-3 (AP-3) mutants in *Drosophila melanogaster*. *PLoS One* 10:e0143026. 10.1371/journal.pone.0143026 26565960 PMC4643998

[B54] Rosencrans W. M. Rajendran M. Bezrukov S. M. Rostovtseva T. K. (2021). VDAC regulation of mitochondrial calcium flux: From channel biophysics to disease. *Cell Calcium* 94:102356. 10.1016/j.ceca.2021.102356 33529977 PMC7914209

[B55] Saha K. Yang J. W. Hofmaier T. Venkatesan S. Steinkellner T. Kudlacek O. et al. (2021). Constitutive endocytosis of the neuronal glutamate transporter excitatory amino acid transporter-3 requires ARFGAP1. *Front. Physiol.* 12:671034. 10.3389/fphys.2021.671034 34040545 PMC8141794

[B56] Schäfer F. Seip N. Maertens B. Block H. Kubicek J. (2015). Purification of GST-tagged proteins. *Methods Enzymol.* 559 127–139. 10.1016/bs.mie.2014.11.005 26096507

[B57] Singh A. Zhi L. Zhang H. (2019). LRRK2 and mitochondria: Recent advances and current views. *Brain Res.* 1702 96–104. 10.1016/j.brainres.2018.06.010 29894679 PMC6281802

[B58] Smith W. W. Pei Z. Jiang H. Dawson V. L. Dawson T. M. Ross C. A. (2006). Kinase activity of mutant LRRK2 mediates neuronal toxicity. *Nat. Neurosci.* 9 1231–1233. 10.1038/nn1776 16980962

[B59] Smith W. W. Pei Z. Jiang H. Moore D. J. Liang Y. West A. B. et al. (2005). Leucine-rich repeat kinase 2 (LRRK2) interacts with parkin, and mutant LRRK2 induces neuronal degeneration. *Proc. Natl. Acad. Sci. U. S. A.* 102 18676–18681. 10.1073/pnas.0508052102 16352719 PMC1317945

[B60] Stafa K. Trancikova A. Webber P. J. Glauser L. West A. B. Moore D. J. (2012). GTPase activity and neuronal toxicity of Parkinson’s disease-associated LRRK2 is regulated by ArfGAP1. *PLoS Genet.* 8:e1002526. 10.1371/journal.pgen.1002526 22363216 PMC3280333

[B61] Stafa K. Tsika E. Moser R. Musso A. Glauser L. Jones A. et al. (2014). Functional interaction of Parkinson’s disease-associated LRRK2 with members of the dynamin GTPase superfamily. *Hum. Mol. Genet.* 23 2055–2077. 10.1093/hmg/ddt600 24282027 PMC3959816

[B62] Steger M. Diez F. Dhekne H. S. Lis P. Nirujogi R. S. Karayel O. et al. (2017). Systematic proteomic analysis of LRRK2-mediated Rab GTPase phosphorylation establishes a connection to ciliogenesis. *eLife* 6:e31012. 10.7554/eLife.31012 29125462 PMC5695910

[B63] Steger M. Tonelli F. Ito G. Davies P. Trost M. Vetter M. et al. (2016). Phosphoproteomics reveals that Parkinson’s disease kinase LRRK2 regulates a subset of Rab GTPases. *eLife* 5:e12813. 10.7554/eLife.12813 26824392 PMC4769169

[B64] Strack R. L. Strongin D. E. Bhattacharyya D. Tao W. Berman A. Broxmeyer H. E. et al. (2008). A noncytotoxic DsRed variant for whole-cell labeling. *Nat. Methods* 5 955–957. 10.1038/nmeth.1264 18953349 PMC4107390

[B65] Sun Y. Vashisht A. A. Tchieu J. Wohlschlegel J. A. Dreier L. (2012). Voltage-dependent anion channels (VDACs) recruit Parkin to defective mitochondria to promote mitochondrial autophagy. *J. Biol. Chem.* 287 40652–40660. 10.1074/jbc.M112.419721 23060438 PMC3504778

[B66] Szklarczyk D. Franceschini A. Wyder S. Forslund K. Heller D. Huerta-Cepas J. et al. (2015). STRING v10: Protein-protein interaction networks, integrated over the tree of life. *Nucleic Acids Res.* 43 D447–D452. 10.1093/nar/gku1003 25352553 PMC4383874

[B67] Taylor T. C. Kahn R. A. Melançon P. (1992). Two distinct members of the ADP-ribosylation factor family of GTP-binding proteins regulate cell-free intra-Golgi transport. *Cell* 70 69–79. 10.1016/0092-8674(92)90534-j 1623523

[B68] Tie H. C. Mahajan D. Chen B. Cheng L. VanDongen A. M. Lu L. (2016). A novel imaging method for quantitative Golgi localization reveals differential intra-Golgi trafficking of secretory cargoes. *Mol. Biol. Cell* 27 848–861. 10.1091/mbc.E15-09-0664 26764092 PMC4803310

[B69] Tyanova S. Temu T. Sinitcyn P. Carlson A. Hein M. Y. Geiger T. et al. (2016). The Perseus computational platform for comprehensive analysis of (prote)omics data. *Nat. Methods* 13 731–740. 10.1038/nmeth.3901 27348712

[B70] Vanni S. Hirose H. Barelli H. Antonny B. Gautier R. (2014). A sub-nanometre view of how membrane curvature and composition modulate lipid packing and protein recruitment. *Nat. Commun.* 5:4916. 10.1038/ncomms5916 25222832

[B71] Wauters L. Versées W. Kortholt A. (2019). Roco proteins: Gtpases with a baroque structure and mechanism. *Int. J. Mol. Sci.* 20:147. 10.3390/ijms20010147 30609797 PMC6337361

[B72] West A. B. Moore D. J. Choi C. Andrabi S. A. Li X. Dikeman D. et al. (2007). Parkinson’s disease-associated mutations in LRRK2 link enhanced GTP-binding and kinase activities to neuronal toxicity. *Hum. Mol. Genet.* 16 223–232. 10.1093/hmg/ddl471 17200152

[B73] Williams E. T. Glauser L. Tsika E. Jiang H. Islam S. Moore D. J. (2018). Parkin mediates the ubiquitination of VPS35 and modulates retromer-dependent endosomal sorting. *Hum. Mol. Genet.* 27 3189–3205. 10.1093/hmg/ddy224 29893854 PMC6121197

[B74] Xiong Y. Coombes C. E. Kilaru A. Li X. Gitler A. D. Bowers W. J. et al. (2010). GTPase activity plays a key role in the pathobiology of LRRK2. *PLoS Genet.* 6:e1000902. 10.1371/journal.pgen.1000902 20386743 PMC2851569

[B75] Xiong Y. Yuan C. Chen R. Dawson T. M. Dawson V. L. (2012). ArfGAP1 is a GTPase activating protein for LRRK2: Reciprocal regulation of ArfGAP1 by LRRK2. *J. Neurosci.* 32 3877–3886. 10.1523/JNEUROSCI.4566-11.2012 22423108 PMC3319331

[B76] Yu S. Roth M. G. (2002). Casein kinase I regulates membrane binding by ARF GAP1. *Mol. Biol. Cell* 13 2559–2570. 10.1091/mbc.e02-04-0189 12181329 PMC117925

[B77] Yun H. J. Park J. Ho D. H. Kim H. Kim C. H. Oh H. et al. (2013). LRRK2 phosphorylates Snapin and inhibits interaction of Snapin with SNAP-25. *Exp. Mol. Med.* 45:e36. 10.1038/emm.2013.68 23949442 PMC3789260

[B78] Zhu H. Tonelli F. Turk M. Prescott A. Alessi D. R. Sun J. (2023). Rab29-dependent asymmetrical activation of leucine-rich repeat kinase 2. *Science* 382 1404–1411. 10.1126/science.adi9926 38127736 PMC10786121

[B79] Zimprich A. Biskup S. Leitner P. Lichtner P. Farrer M. Lincoln S. et al. (2004). Mutations in LRRK2 cause autosomal-dominant parkinsonism with pleomorphic pathology. *Neuron* 44 601–607. 10.1016/j.neuron.2004.11.005 15541309

