# Supplementary figures and images for: LRRK2 regulates ArfGAP1 membrane localization, activity and neuronal integrity via phosphorylation within its lipid-sensing ALPS2 motif
Source: Front Mol Neurosci. 2026 Apr 21;19:1786336. doi: 10.3389/fnmol.2026.1786336 (PMC13139335; doi:10.3389/fnmol.2026.1786336)

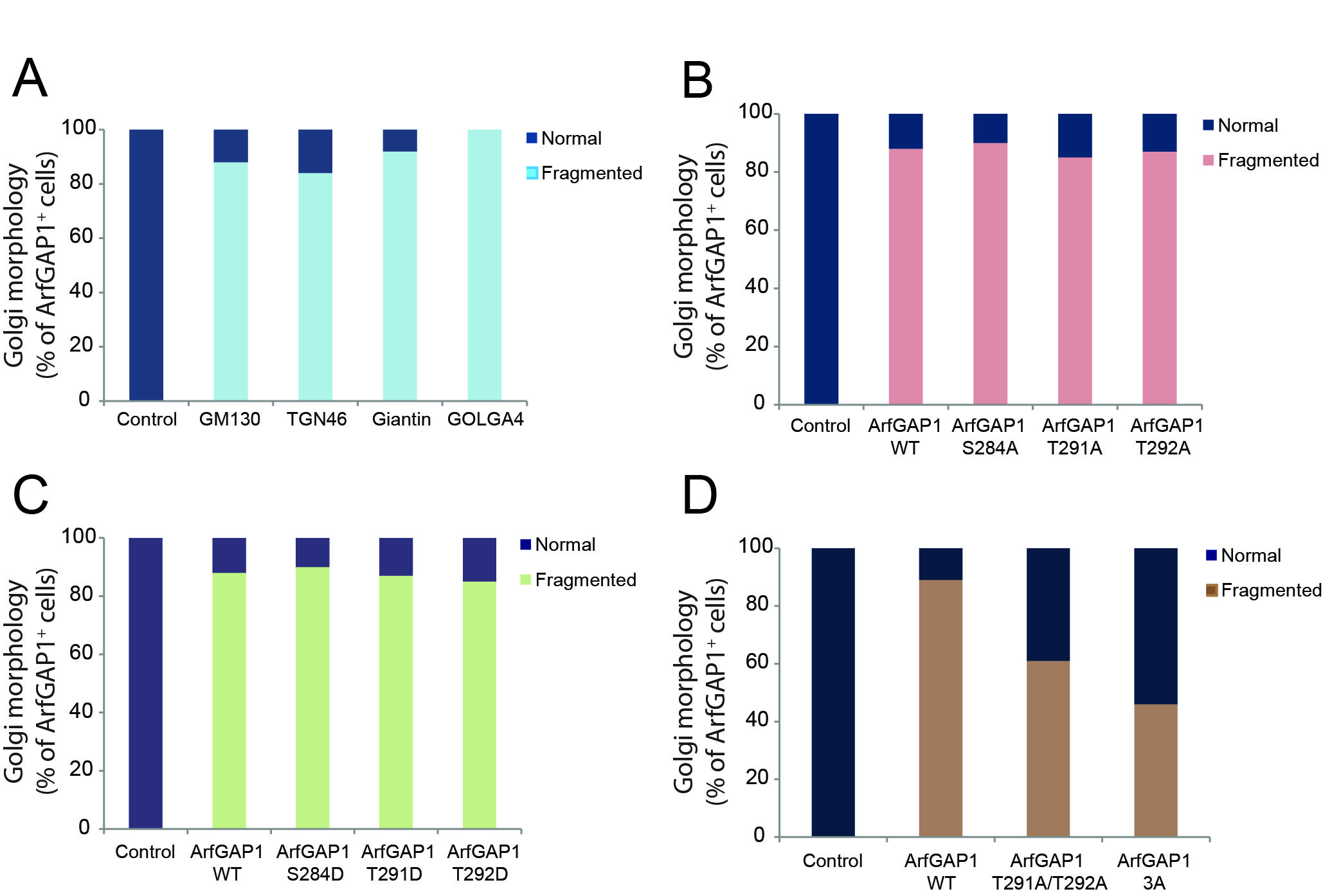

Supplement: Supplementary Figure S1 — Impact of single phosphorylation mutants on ArfGAP1-induced Golgi fragmentation in neural cells. (A) SH-SY5Y cells transiently expressing YFP-tagged WT ArfGAP1 or empty vector were fixed and subjected to immunofluorescence with antibodies to different Golgi membrane markers (GM130, TGN46, Giantin, GOLGA4). Quantitation of Golgi morphology in ArfGAP1-positive cells reveals similar levels of Golgi fragmentation between Golgi markers (90–100% cells). (B,C) Quantitation of Golgi fragmentation induced by overexpression of WT or single phospho-mutants of YFP-ArfGAP1 in SH-SY5Y cells. Single phospho-null or phospho-mimic mutants induce similar Golgi fragmentation levels to WT ArfGAP1 (∼85% cells). (D) Golgi fragmentation induced by ArfGAP1 double (T291A/T292A) or triple (3A) phospho-null mutants indicates partial Golgi fragmentation of double mutants (∼65% cells) or 3A mutant (∼45% cells) compared to WT ArfGAP1 (∼85% cells). In each graph, bars represent the percentage of cells with normal or fragmented Golgi from a representative experiment. [file Image_1.jpeg]

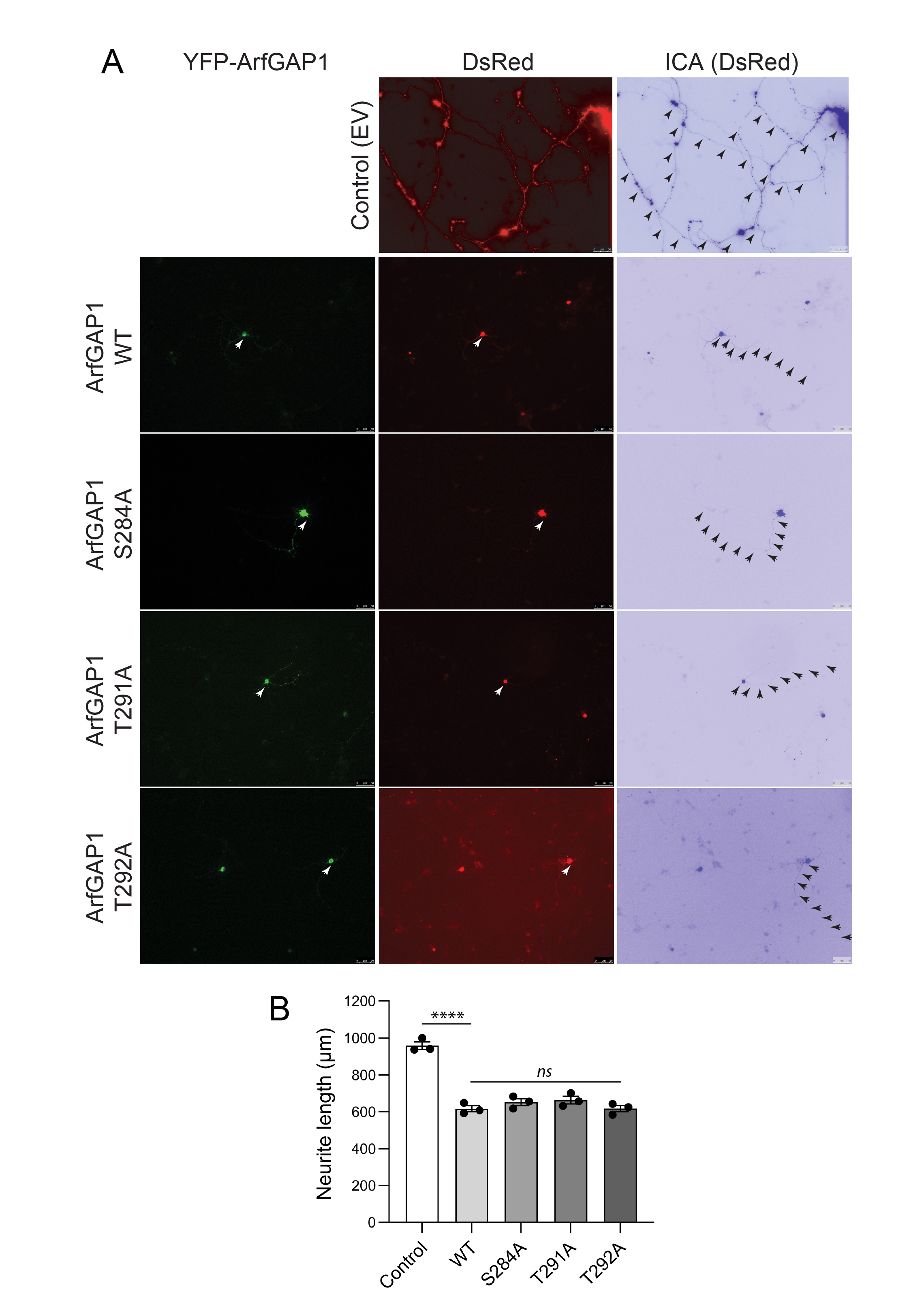

Supplement: Supplementary Figure S2 — Impact of single phospho-null mutants on ArfGAP1-induced inhibition of neurite outgrowth. (A) Rat primary cortical neurons were co-transfected at DIV3 with YFP-ArfGAP1 (WT, S284A, T291A or T292A) or empty vector and DsRed-Max-N1 plasmids, and fixed at DIV6 for confocal fluorescence microscopy analysis. Fluorescent images reveal the co-labeling of cortical neurons with YFP-ArfGAP1 (green) and DsRed (red), with the DsRed images pseudo colored (ICA) to enhance the contrast of neuritic processes. Neuronal soma (white arrows) and axonal processes (black arrowheads) are indicated. Scale bars: 50 μm. (B) Quantitative analysis of DsRed-positive axon length in YFP-ArfGAP1-positive neurons or control neurons (empty vector) is shown. Bars represent the mean ± SEM axon length (in μm) from 90 to 120 double DsRed-/YFP-ArfGAP1-positive neurons, or single DsRed-positive neurons (control), across three independent experiments/cultures (n = 3). ****P < 0.0001 as indicated compared to ArfGAP1-WT by one-way ANOVA with Tukey’s multiple comparisons test. ns, non-significant. [file Image_2.jpeg]

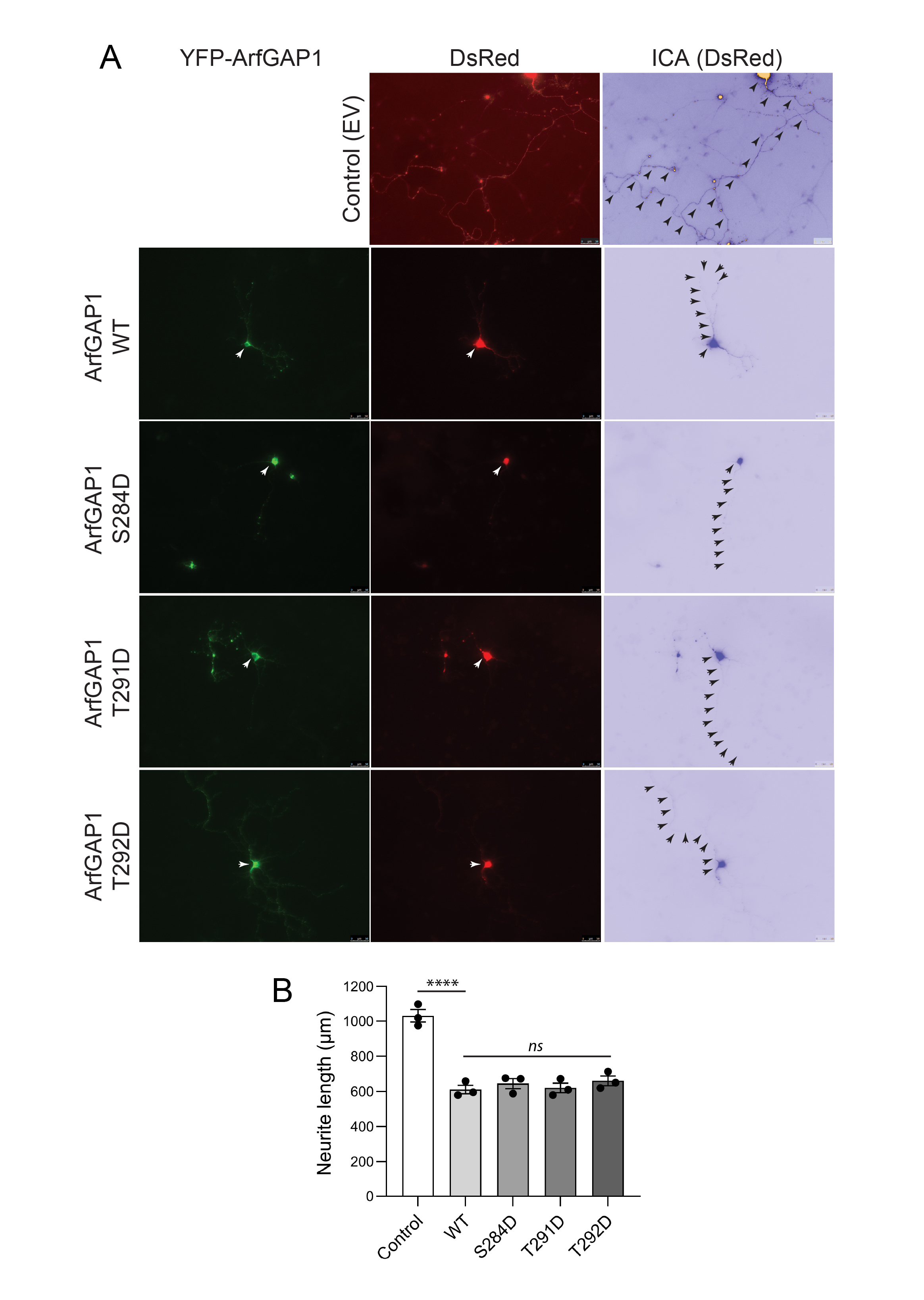

Supplement: Supplementary Figure S3 — Impact of single phospho-mimic mutants on ArfGAP1-induced inhibition of neurite outgrowth. (A) Rat primary cortical neurons were co-transfected at DIV3 with YFP-ArfGAP1 (WT, S284D, T291D or T292D) or empty vector and DsRed-Max-N1 plasmids, and fixed at DIV6 for confocal fluorescence microscopy analysis. Fluorescent images reveal the co-labeling of cortical neurons with YFP-ArfGAP1 (green) and DsRed (red), with the DsRed images pseudo colored (ICA) to enhance the contrast of neuritic processes. Neuronal soma (white arrows) and axonal processes (black arrowheads) are indicated. Scale bars: 50 μm. (B) Quantitative analysis of DsRed-positive axon length in YFP-ArfGAP1-positive neurons or control neurons (empty vector) is shown. Bars represent the mean ± SEM axon length (in μm) from 90 to 120 double DsRed-/YFP-ArfGAP1-positive neurons, or single DsRed-positive neurons (control), across three independent experiments/cultures (n = 3). ****P < 0.0001 as indicated compared to ArfGAP1-WT by one-way ANOVA with Tukey’s multiple comparisons test. ns, non-significant. [file Image_3.jpeg]
